# Supplementary material for: RcsF-independent mechanisms of signaling within the Rcs phosphorelay
Source: PLoS Genet. 2024 Dec 26;20(12):e1011408. doi: 10.1371/journal.pgen.1011408 (PMC11709261; doi:10.1371/journal.pgen.1011408)
Supplement: S1 Table — (DOCX) [file pgen.1011408.s001.docx]

**Table S1: List of strains used in this study**

Strains were constructed by recombineering or P1 transduction with selectable markers (Table S1). Recombineering was done in strains carrying a chromosomal mini-λ Red system (*miniλ::tet*) or a plasmid-borne Red system (pSIM27). Some strains were generated by direct P1 transduction from the corresponding mutant strains in the Keio collection [1].

| **Name** | **Genotype** | **Method of construction or reference** |
| --- | --- | --- |
| MG1655 | Wild-type *E. coli* K-12 | Lab collection |
| BTH101 | *F-, cya-99, araD139, galE15, galK16, rpsL1 (Str^r^), hsdR2, mcrA1, mcrB1* | [2] |
| NEB DH5-alpha F'IQ | F´ *proA^+^B^+^ lacI^q^ ∆(lacZ)M15 zzf::Tn10*(Tet^R^)*/ fhuA2∆(argF-lacZ)U169 phoA glnV44 Φ80Δ(lacZ)M15 gyrA96 recA1 relA1 endA1 thi-1 hsdR17* | New England Biolabs |
| SG20382 | *rcsB11*::Tn10 (*tet^r^*) | [3] |
| DH300 | P*_rprA142-_lacZ* | [4] |
| DH311 | P*_rprA142-_lacZ*, *rcsB311*::*kan* | [4] |
| DH339 | P*_rprA142_*-*lacZ*, *yojN*::*kan* (*rcsD542*) | [5] |
| DH375 | P*_rprA142_*-*lacZ*, *rcsC C111A C154A atoS*::*kan* | DH300 + P1 (NM344a)^#^ |
| DJ480 | MG1655 *lacX74* | [6] |
| TKC | *tetA, cat, kan* | [7] |
| NC397 | *W3110 pglΔ8 gal490 λ cI857(cro-bioA)Δ lacI^o^ <>kan-Ter<>cat sacB <>lacZYA* | [8] |
| HK307 | MC1000 *dsbA::kan* | Beckwith lab |
| EC251 | MG1655 | [9] |
| EC855 | MG1655 *ftsE::kan* | Weiss lab |
| EC1215 | *∆ftsEX<>frt* | [9] |
| NM7 | *∆rcsF12*::*cat*-*sacB* | [10] |
| NM300 | DJ480 *mini-λ-tet* | [11] |
| NM338 | DJ480 *rcsC C111A- cat-sacB* | NM300 + linear transformation^#^ |
| NM340 | DJ480 *rcsC C111A* | NM338 + single-stranded Cys111Ala replacement primer^#^ |
| NM344a | DJ480 *rcsC C111A C154A with atoS*::*kan* | NM355 + linear transformation^#^ |
| NM350 | DJ480 *rcsC C111A with atoS*::*kan* | NM340 + linear transformation^#^ |
| NM355 | DJ480 *∆rcsC154-atoS::cat* | NM300 linear transformation^#^ |
| NM358 | DJ480 *mini-λ-tet, rcsB311::kan* | NM300 + P1 (DH311) |
| NM364 | DJ480 *mini-λ-tet, rcsB311::kan, ∆wza-∆cpsB::zeo* | NM358 electroporated with NM1201 PCR product (cpsB-zeo.R and wza-zeo.F) |
| NM1201 | MG1655 *ybeW::zeo* | [12] |
| EAW1 | *rcsB11*::Tn10, *cya* | [10] |
| EAW2 | *rcsC32*::Tn10, *cya* | [10] |
| EAW4 | *∆rcsF12*::*cat*-*sacB*, *cya* | [10] |
| EAW8 | *∆araBAD*::P*_rprA142_*-mCherry, *∆araE*p P_CP6_::*gent::*P*_cp18_*-*araE* | [10] |
| EAW18 | *∆araBAD*::P*_rprA142_*-mCherry, *rcsC*::Tn10, *∆araE*p P_cp6_*gent*::P_cp18_-*araE* | [10] |
| EAW19 | *∆araBAD*::P*_rprA142_*-mCherry, *rcsD*541(::FRT), *∆araEp* P*_cp6_gent::*P*_cp18_-araE* | [10] |
| EAW25 | *∆araBAD*::P*_rprA142_*-mCherry, *∆araE*p P_CP6_::*gent::*P*_cp18_*-*araE, ∆wza-∆cpsB::zeo* | EAW8 + P1 from NM364 (selected for *∆wza-∆cpsB::zeo*) |
| EAW31 | *∆araBAD*::P*_rprA142_*-mCherry, *∆araE*p P_cp6_*gent*::P_cp18_-*araE, rcsB*::*kan* | [10] |
| EAW32 | *∆araBAD*::P*_rprA142_*-mCherry, *∆araE*p P_cp6_*gent*::P_cp18_-*araE*, *∆rcsF12*::*cat*-*sacB* | [10] |
| EAW34 | *∆araBAD*::P*_rprA142_*-mCherry, *∆araE*p P_CP6_::*gent::*P*_cp18_*-*araE, ∆wza-∆cpsB::zeo*, ∆*rcsF12::cat-sacB* | EAW25 + P1 (NM7) |
| EAW70 | *∆araBAD*::P*_rprA142_*-mCherry, *∆araE*p P_cp6_*gent*::P_cp18_-*araE*, *rcsC*∆*peri* (*rcsC∆48-314)* | [10] |
| EAW72 | *∆araBAD*::P*_rprA142_*-mCherry, *∆araE*p P_cp6_*gent*::P_cp18_-*araE*, *rcsC_1-19_-malF_2-59_-rcsC_334-C_* | [10] |
| EAW 88 | *ΔaraBAD*::P*_rprA142_*-mCherry, *ΔaraE*p P_cp6_*gent*::P_cp18_-*araE*, *ΔrcsC88::kan-araC-kid* | [10] |
| EAW 91 | *ΔaraBAD*::P*_rprA142_*-mCherry, *ΔaraE*p P_cp6_*gent*::P_cp18_-*araE*, *ΔrcsC91* | [10] |
| EAW120 | *∆araBAD*::P*_rprA142_*-mCherry, *∆araE*p P_cp6_*gent*::P_cp18_-*araE*, *rcsD841** | [10] |
| EAW121 | *∆araBAD*::P*_rprA142_*-mCherry, *∆araE*p P_cp6_*gent*::P_cp18_-*araE*, *rcsDT411A* | [10] |
| EAW62 | *∆araBAD*::P*_rprA142_*-mCherry, *∆araE*p P_CP6_::*gent::*P*_cp18_*-*araE*, *dsbA::kan* | EAW8 + P1 (HK307) |
| EAW63 | *∆araBAD*::P*_rprA142_*-mCherry, *rcsC*::Tn10, *∆araE*p P_cp6_*gent*::P_cp18_-*araE*, *dsbA::kan* | EAW18 + P1 (HK307) |
| EAW67 | *∆araBAD*::P*_rprA142_*-mCherry, *∆araE*p P_CP6_::*gent::*P*_cp18_*-*araE*, *dsbA::kan*, ∆*rcsF12::cat-sacB* | EAW62 + P1 (NM7) |
| EAW74 | *∆araBAD*::P*_rprA142_*-mCherry, *∆araE*p P_cp6_*gent*::P_cp18_-*araE*, *rcsC∆peri* (*rcsC∆48-314*) *dsbA::kan* | EAW70 + P1 (HK307) |
| EAW90 | *∆araBAD*::P_rprA142_-mCherry, *∆araE*p P_cp6_*gent*::P_cp18_-*araE*, *rcsD541(::FRT)*, *∆igaA*::*kan-araC-kid* | [10] |
| AP11 | *∆araBAD*::P*_rprA142_*-mCherry, *∆araE*p P_CP6_::*gent::*P*_cp18_*-*araE*, ∆*dsbA* | EAW62 + pCP20 |
| AP12 | *∆araBAD*::P*_rprA142_*-mCherry, *∆araE*p P_CP6_::*gent::*P*_cp18_*-*araE*, ∆*dsbA*, *rcsB311::kan* | AP11 + P1 (DH311) |
| AP13 | *∆araBAD*::P*_rprA142_*-mCherry, *∆araE*p P_CP6_::*gent::*P*_cp18_*-*araE*, ∆*dsbA*, *yojN::kan (rcsD542)* | AP11 + P1 (DH339) |
| AP14 | *∆araBAD*::P*_rprA142_*-mCherry, *∆araE*p P_cp6_*gent*::P_cp18_-*araE*, *rcsDT411A*, *dsbA::kan* | EAW121 + P1 (HK307) |
| AP41 | *∆araBAD*::P*_rprA142_*-mCherry, *∆araE*p P_CP6_::*gent::*P*_cp18_*-*araE, drpB::kan* | EAW8 + P1 (Keio JW1946) |
| AP46 | *∆araBAD*::P*_rprA142_*-mCherry, *∆araE*p P_CP6_::*gent::*P*_cp18_*-*araE, djlA::kan* | EAW8 + P1 (Keio JW0054) |
| AP50 | *∆araBAD*::P*_rprA142_*-mCherry, *∆araE*p P_CP6_::*gent::*P*_cp18_*-*araE*, *rcsB11::Tn10 (tetr)* | EAW8 + P1 (SG20382) |
| AP51 | ∆*araBAD*::P*_rprA142_*-mCherry, ∆*araE*p P_CP6_::*gent::*P*_cp18_*-*araE, rcsF::kan* | EAW8 + P1 (Keio JW0192) |
| AP57 | *dsbA::kan, cya* | BTH101 + P1 (HK307) |
| AP58 | *∆dsbA, cya* | AP57 + pcp20 |
| AP61 | *djlA::kan, cya* | BTH101 + P1 (Keio JW0054) |
| AP63 | *∆djlA, cya* | AP61 + pcp20 |
| AP71 | *∆araBAD*::P*_rprA142_*-mCherry, ∆*araE*p P_CP6_::*gent::*P*_cp18_*-*araE*, ∆*dsbA, drpB::kan* | AP11 + P1 (Keio JW1946) |
| AP72 | *∆araBAD*::P*_rprA142_*-mCherry, *∆araE*p P_CP6_::*gent::*P*_cp18_*-*araE*, ∆*dsbA*, *djlA::kan* | AP11 + P1 (Keio JW0054) |
| AP113 | *∆araBAD*::P*_rprA142_*-mCherry, *∆araE*p P_CP6_::*gent::*P*_cp18_*-*araE, pspC::kan* | EAW8 + P1 (Keio JW1299) |
| AP114 | *∆araBAD*::P*_rprA142_*-mCherry, *∆araE*p P_CP6_::*gent::*P*_cp18_*-*araE, pspF::kan* | EAW8 + P1 (Keio JW1296) |
| AP154 | *∆araBAD*::P*_rprA142_*-mCherry, *∆araE*p P_CP6_::*gent::*P*_cp18_*-*araE, ftsE::kan* | EAW8 + P1 (EC855) |
| AP158 | *rcsB311*::*kan* | EC251 + P1 (DH311) |
| AP159 | *∆ftsEX<>frt, rcsB311*::*kan* | EC1215 + P1 (DH311) |
| AP168 | *∆araBAD*::P_rprA142_-mCherry, *∆araE*p P_cp6_*gent*::P_cp18_-*araE*, *rcsD541(::FRT)*, *∆igaA::igaA C404S C424S C498S C504S* | EAW90 recombination with PCR product of pEAW1C4S template using oligos EAW213 and EAW214 |
| AP169 | *∆araBAD*::P*_rprA142_*-mCherry, *rcsD541(::FRT)*, *∆araEp* P*_cp6_gent::*P*_cp18_-araE*, *dsbA::kan* | EAW19 + P1 (EAW62) |
| AP172 | *∆araBAD*::P*_rprA142_*-mCherry, *∆araE*p P_CP6_::*gent::*P*_cp18_*-*araE*, *rcsC C111A C154A with atoS::kan* | EAW8 + P1 (DH375) |
| AP173 | *∆araBAD*::P*_rprA142_*-mCherry, *∆araE*p P_CP6_::*gent::*P*_cp18_*-*araE*, *rcsC C111A C154A with atoS::kan, ∆dsbA* | AP11 + P1 (DH375) |
| AP 200 | *∆araBAD*::P*_rprA142_*-mCherry, *∆araE*p P_CP6_::*gent::*P*_cp18_*-*araE, barA::kan* | EAW8 + P1 (Keio JW2757) |
| AP 201 | *ΔaraBAD*::P*_rprA142_*-mCherry, *ΔaraE*p P_cp6_*gent*::P_cp18_-*araE*, *ΔrcsC91, barA::kan* | EAW91 + P1 (Keio JW2757) |
| AP 202 | *∆araBAD*::P*_rprA142_*-mCherry, *rcsD*541(::FRT), *∆araEp* P*_cp6_gent::*P*_cp18_-araE, barA::kan* | EAW19 + P1 (Keio JW2757) |
| AP 205 | *∆araBAD*::P*_rprA142_*-mCherry, *∆araE*p P_CP6_::*gent::*P*_cp18_*-*araE*, *rcsB11::Tn10 (tetr), barA::kan* | AP50 + P1 (Keio JW2757) |
| AP 206 | *∆araBAD*::P*_rprA142_*-mCherry, *∆araE*p P_CP6_::*gent::*P*_cp18_*-*araE*, ∆*dsbA, barA::kan* | AP11 + P1 (Keio JW2757) |

**^#^Construction of NM344a:**

NM344a was constructed in two steps. In the first step, the region between *atoS* and *rcsCcys154* in NM300 was replaced by recombineering a *cat* resistance cassette using the primers Δ*atoS-rcsC154.CmF* and Δ*atoS-rcsC154.CmR* and the TKC strain used as a template. This generated NM355. In a second parallel step, NM338 was generated by recombineering the PCR product (primers Cys111-ala_cat and Cys111-ala_sacB) from NC397 into NM300. Next, NM340 was generated by linear transformation of the single-stranded oligo Cys111Ala replacement primer into NM338. In this strain NM340, a *kan* resistance cassette was then inserted between *rcsCcys111* and *atoS* using primers RcsC-KAN-AtoS.F and RcsC-KAN-AtoS.R. This generated strain NM350. A 2-kb fragment from the *kan* cassette to the rcsC154 nucleotide was amplified from NM350 using the primers atoS_RcsCys154 (containing the Cys154Ala mutation) and RcsC-KAN-AtoS.R was used to transform NM355. This generated strain NM344a.

**References:**

1. Baba T, Ara T, Hasegawa M, Takai Y, Okumura Y, Baba M, et al. Construction of Escherichia coli K-12 in-frame, single-gene knockout mutants: the Keio collection. Mol Syst Biol. 2006;2:2006.0008. Epub 20060221. doi: 10.1038/msb4100050. PubMed PMID: 16738554; PubMed Central PMCID: PMCPMC1681482.

2. Karimova G, Pidoux J, Ullmann A, Ladant D. A bacterial two-hybrid system based on a reconstituted signal transduction pathway. Proc Natl Acad Sci U S A. 1998;95(10):5752-6. doi: 10.1073/pnas.95.10.5752. PubMed PMID: 9576956; PubMed Central PMCID: PMCPMC20451.

3. Brill JA, Quinlan-Walshe C, Gottesman S. Fine-structure mapping and identification of two regulators of capsule synthesis in Escherichia coli K-12. J Bacteriol. 1988;170(6):2599-611. doi: 10.1128/jb.170.6.2599-2611.1988. PubMed PMID: 2836365; PubMed Central PMCID: PMCPMC211177.

4. Majdalani N, Hernandez D, Gottesman S. Regulation and mode of action of the second small RNA activator of RpoS translation, RprA. Mol Microbiol. 2002;46(3):813-26. doi: 10.1046/j.1365-2958.2002.03203.x. PubMed PMID: 12410838.

5. Majdalani N, Heck M, Stout V, Gottesman S. Role of RcsF in signaling to the Rcs phosphorelay pathway in Escherichia coli. J Bacteriol. 2005;187(19):6770-8. doi: 10.1128/jb.187.19.6770-6778.2005. PubMed PMID: 16166540; PubMed Central PMCID: PMCPMC1251585.

6. Cabrera JE, Jin DJ. Growth phase and growth rate regulation of the rapA gene, encoding the RNA polymerase-associated protein RapA in Escherichia coli. J Bacteriol. 2001;183(20):6126-34. doi: 10.1128/jb.183.20.6126-6134.2001. PubMed PMID: 11567013; PubMed Central PMCID: PMCPMC99692.

7. Sharan SK, Thomason LC, Kuznetsov SG, Court DL. Recombineering: a homologous recombination-based method of genetic engineering. Nat Protoc. 2009;4(2):206-23. doi: 10.1038/nprot.2008.227. PubMed PMID: 19180090; PubMed Central PMCID: PMCPMC2790811.

8. Svenningsen SL, Costantino N, Court DL, Adhya S. On the role of Cro in lambda prophage induction. Proc Natl Acad Sci U S A. 2005;102(12):4465-9. Epub 20050223. doi: 10.1073/pnas.0409839102. PubMed PMID: 15728734; PubMed Central PMCID: PMCPMC555511.

9. Yahashiri A, Babor JT, Anwar AL, Bezy RP, Piette EW, Arends SJR, et al. DrpB (YedR) Is a Nonessential Cell Division Protein in Escherichia coli. J Bacteriol. 2020;202(23). Epub 20201104. doi: 10.1128/jb.00284-20. PubMed PMID: 32900831; PubMed Central PMCID: PMCPMC7648144.

10. Wall EA, Majdalani N, Gottesman S. IgaA negatively regulates the Rcs Phosphorelay via contact with the RcsD Phosphotransfer Protein. PLoS Genet. 2020;16(7):e1008610. Epub 20200727. doi: 10.1371/journal.pgen.1008610. PubMed PMID: 32716926; PubMed Central PMCID: PMCPMC7418988.

11. Thompson KM, Rhodius VA, Gottesman S. SigmaE regulates and is regulated by a small RNA in Escherichia coli. J Bacteriol. 2007;189(11):4243-56. Epub 20070406. doi: 10.1128/jb.00020-07. PubMed PMID: 17416652; PubMed Central PMCID: PMCPMC1913397.

12. Battesti A, Tsegaye YM, Packer DG, Majdalani N, Gottesman S. H-NS regulation of IraD and IraM antiadaptors for control of RpoS degradation. J Bacteriol. 2012;194(10):2470-8. Epub 20120309. doi: 10.1128/jb.00132-12. PubMed PMID: 22408168; PubMed Central PMCID: PMCPMC3347191.
