# Supplementary material for: RcsF-independent mechanisms of signaling within the Rcs phosphorelay
Source: PLoS Genet. 2024 Dec 26;20(12):e1011408. doi: 10.1371/journal.pgen.1011408 (PMC11709261; doi:10.1371/journal.pgen.1011408)
Supplement: S2 Table — (DOCX) [file pgen.1011408.s002.docx]

**Table S2: List of plasmids used in this study**

Plasmids were constructed by the Gibson assembly method using the In-fusion HD Cloning kit (Takara Bio USA) [1]. Site-directed mutagenesis (SDM) in the genes was carried out using the QuikChange Site-directed mutagenesis kit (Agilent).

| **Name** | **Description** | **Method of construction/ Reference** |
| --- | --- | --- |
| pBAD24 | pBR322-based vector for protein expression driven by the *araBAD* operon promoter (arabinose inducible) (Amp^r^) | [2] |
| pBAD33 | pACYC184-based vector for protein expression driven by the *araBAD* operon promoter (arabinose inducible) (Chl^r^) | [2] |
| pCP20 | Plasmid with temperature-sensitive origin of replication, encoding the FLP recombinase | [3] |
| pUT18 | Vector encoding the Cya T18 fragment under lac promoter control (Amp^r^) | [4] |
| pKNT25 | Vector encoding the Cya T18 fragment under lac promoter control (Kan^r^) | [5] |
| pSIM27 | Plasmid with temperature-sensitive origin of replication, encoding l-Red cI857, gam-beta-exo | https://ncifrederick.cancer.gov/recombineering/strains-plasmids-and-primers |
| pEAW1 | IgaA with T18 tag at C-terminal cloned in pUT18 | [6] |
| pEAW1cyt1 | IgaA *∆*36-181 with T18 tag at C-terminal cloned in pUT18 | [6] |
| pEAW1peri | IgaA *∆*384-649 with T18 tag at C-terminal cloned in pUT18 | [6] |
| pEAW2 | IgaA with T25 tag at C-terminal cloned in pKNT25 | [6] |
| pEAW6 | RcsC with T25 tag at C-terminal cloned in pKNT25 | [6] |
| pEAW7 | RcsD with T18 tag at C-terminal cloned in pUT18 | [6] |
| pEAW8 | RcsD with T25 tag at C-terminal cloned in pKNT25 | [6] |
| pEAW8peri | RcsD *∆*45-304 with T25 tag at C-terminal cloned in pKNT25 | [6] |
| pEAW8T | RcsD T411A with T25 tag at C-terminal cloned in pKNT25 | [6] |
| pEAW11 | RcsD cloned in pBAD24 | [6] |
| pEAW11T | RcsD T411A cloned in pBAD24 | [6] |
| pPSG961 | DjlA cloned in pBAD33 | [7] |
| pDSW1977 | DrpB cloned in pBAD33 | [8] |
| pBR-plac | pBR322 derivative plasmid for sRNA overexpression under an artificial P_lac_ promoter | [9] |
| p-rseX | rseX sRNA cloned in pBR-plac | [10] |
| p-spf | spot42 sRNA cloned in pBR-plac | [10] |
| pNM654 | IgaA cloned in pBAD24 | IgaA amplified from genomic DNA (yrfF_pBAD24F and R); pBAD24 digested with *EcoRI* and *HindIII* |
| pNM656 | IgaA C425S cloned in pBAD24 | pNM654 template with primers yrfF Cys425Ser.F and Cys425Ser.R (SDM) |
| pNM665 | IgaA C425S C498S C504S cloned in pBAD24 | pNM656 template with primers yrfF Cys498-504Ser.F and Cys498-504Ser.R (SDM) |
| pNM671 | IgaA C404S C425S C498S C504S cloned in pBAD24 | pNM665 template with primers yrfF Cys404Ser.F and Cys404Ser.R (SDM) |
| pAP101 | IgaA *∆*36-181 ∆263-329 with T18 tag at C-terminal cloned in pUT18 | pEAW1cyt1 template with primers EW209 and EW210 |
| pAP102 | IgaA C404S C425S with T18 tag at C-terminal cloned in pUT18 | pAP104 template with primers AP691 and AP692 (SDM) |
| pAP103 | IgaA C498S C504S with T18 tag at C-terminal cloned in pUT18 | pAP105 template with primers AP587 and AP588 (SDM) |
| pAP104 | IgaA C404S with T18 tag at C-terminal cloned in pUT18 | pEAW1 template with primers AP351 and AP352 (SDM) |
| pAP105 | IgaA C498S with T18 tag at C-terminal cloned in pUT18 | pEAW1 template with primers AP693 and AP694 (SDM) |
| pEAW1C4S | IgaA C404S C425S C498S C504S (C4S) with T18 tag at C-terminal cloned in pUT18 | Insert from pNM671 (EW6 and EW7); pUT18 linearized with EW1 and EW2 |
| pAP1401 | IgaA with T18 tag at C-terminal and DjlA cloned downstream under the same promoter in pUT18 | Insert from gBlock AP_GJ1; pEAW1 linearized with AP375 and AP376 |
| pAP1402 | IgaA with T18 tag at C-terminal and DjlA H233Q cloned downstream under the same promoter in pUT18 | pAP1401 template with primers AP459 and AP460 (SDM) |
| pAP1403 | IgaA with T18 tag at C-terminal and DjlA ∆1-31 (∆TM) cloned downstream under the same promoter in pUT18 | pAP1401 template with primers AP495 and AP496 (SDM) |
| pAP1404 | IgaA with T18 tag at C-terminal and DjlA TM_MalF_ cloned downstream under the same promoter in pUT18 | Insert from gBlock AP_GJMF; pEAW1 linearized with AP375 and AP376 |
| pAP407 | DrpB with T18 tag at C-terminal cloned in pUT18 | Insert from pDSW1977 (AP241 and AP242); pUT18 linearized with EW1 and EW2 |
| pAP408 | DrpB with T25 tag at C-terminal cloned in pKNT25 | Insert from pDSW1977 (AP241 and AP242); pkNT25 linearized with EW1 and EW2 |
| pAP804 | RcsD T411A ∆45-304 with T25 tag at C-terminal cloned in pKNT25 | pEAW8peri template with primers T411A F and T411A R (SDM) |
| pAP3301 | DrpB ∆48-59 cloned in pBAD33 | pDSW1977 template with primers AP321 and AP322 (SDM) |
| pAP3304 | DrpB C29A cloned in pBAD33 | pDSW1977 template with primers AP323 and AP324 (SDM) |
| pAP3305 | DrpB R38F cloned in pBAD33 | pDSW1977 template with primers AP325 and AP326 (SDM) |
| pAP3306 | DrpB G83A cloned in pBAD33 | pDSW1977 template with primers AP327 and AP328 (SDM) |
| pAP3307 | DrpB T89A cloned in pBAD33 | pDSW1977 template with primers AP329 and AP330 (SDM) |
| pAP3311 | DjlA ∆1-31 (∆TM) cloned in pBAD33 | pPSG961 template with primers AP563 and AP564 |
| pAP3312 | DjlA TM_MalF_ cloned in pBAD33 | Insert from gBlock AP_GJMF (AP565 and AP566); pBAD33 linearized with AP559 and AP560 |
| pAP3315 | DjlA H233Q cloned in pBAD33 | pPSG961 template with primers AP459 and AP460 (SDM) |
| pAP3325 | YmgB/AriR cloned in pBAD33 | Insert from gBlock AP_GymgB; pBAD33 linearized with AP559 and AP560 |
| pAP3327 | YihA cloned in pBAD33 | Insert from gBlock AP_GyihA; pBAD33 linearized with AP559 and AP560 |
| pAP3340 | RcsF cloned in pBAD33 | Insert from gBlock AP_GF33; pBAD33 linearized with AP559 and AP560 |
| pAP3341 | RcsF S17D M18Q cloned in pBAD33 | pAP3340 template with primers AP501 and AP502 (SDM) |

**References:**

1. Gibson DG, Young L, Chuang RY, Venter JC, Hutchison CA, 3rd, Smith HO. Enzymatic assembly of DNA molecules up to several hundred kilobases. Nat Methods. 2009;6(5):343-5. Epub 20090412. doi: 10.1038/nmeth.1318. PubMed PMID: 19363495.

2. Guzman LM, Belin D, Carson MJ, Beckwith J. Tight regulation, modulation, and high-level expression by vectors containing the arabinose PBAD promoter. J Bacteriol. 1995;177(14):4121-30. doi: 10.1128/jb.177.14.4121-4130.1995. PubMed PMID: 7608087; PubMed Central PMCID: PMCPMC177145.

3. Cherepanov PP, Wackernagel W. Gene disruption in Escherichia coli: TcR and KmR cassettes with the option of Flp-catalyzed excision of the antibiotic-resistance determinant. Gene. 1995;158(1):9-14. doi: 10.1016/0378-1119(95)00193-a. PubMed PMID: 7789817.

4. Karimova G, Ullmann A, Ladant D. Protein-protein interaction between Bacillus stearothermophilus tyrosyl-tRNA synthetase subdomains revealed by a bacterial two-hybrid system. J Mol Microbiol Biotechnol. 2001;3(1):73-82. PubMed PMID: 11200232.

5. Karimova G, Dautin N, Ladant D. Interaction network among Escherichia coli membrane proteins involved in cell division as revealed by bacterial two-hybrid analysis. J Bacteriol. 2005;187(7):2233-43. doi: 10.1128/jb.187.7.2233-2243.2005. PubMed PMID: 15774864; PubMed Central PMCID: PMCPMC1065216.

6. Wall EA, Majdalani N, Gottesman S. IgaA negatively regulates the Rcs Phosphorelay via contact with the RcsD Phosphotransfer Protein. PLoS Genet. 2020;16(7):e1008610. Epub 20200727. doi: 10.1371/journal.pgen.1008610. PubMed PMID: 32716926; PubMed Central PMCID: PMCPMC7418988.

7. Clarke DJ, Holland IB, Jacq A. Point mutations in the transmembrane domain of DjlA, a membrane-linked DnaJ-like protein, abolish its function in promoting colanic acid production via the Rcs signal transduction pathway. Mol Microbiol. 1997;25(5):933-44. doi: 10.1111/j.1365-2958.1997.mmi528.x. PubMed PMID: 9364918.

8. Yahashiri A, Babor JT, Anwar AL, Bezy RP, Piette EW, Arends SJR, et al. DrpB (YedR) Is a Nonessential Cell Division Protein in Escherichia coli. J Bacteriol. 2020;202(23). Epub 20201104. doi: 10.1128/jb.00284-20. PubMed PMID: 32900831; PubMed Central PMCID: PMCPMC7648144.

9. Guillier M, Gottesman S. Remodelling of the Escherichia coli outer membrane by two small regulatory RNAs. Mol Microbiol. 2006;59(1):231-47. doi: 10.1111/j.1365-2958.2005.04929.x. PubMed PMID: 16359331.

10. Mandin P, Gottesman S. Integrating anaerobic/aerobic sensing and the general stress response through the ArcZ small RNA. Embo j. 2010;29(18):3094-107. Epub 20100803. doi: 10.1038/emboj.2010.179. PubMed PMID: 20683441; PubMed Central PMCID: PMCPMC2944060.
