# Supplementary material for: RcsF-independent mechanisms of signaling within the Rcs phosphorelay
Source: PLoS Genet. 2024 Dec 26;20(12):e1011408. doi: 10.1371/journal.pgen.1011408 (PMC11709261; doi:10.1371/journal.pgen.1011408)
Supplement: S3 Table — (DOCX) [file pgen.1011408.s003.docx]

**Table S3: List of primers used in this study**

| **Name** | **Sequence (5'-3')** |
| --- | --- |
| EW1 | AAT CAT GGT CAT AGC TGT TTC CTG TGT GAA ATT G |
| EW2 | AGC TTG CAT GCC TGC AGG TCG AC |
| EW6 | GCT ATG ACC ATG ATT AGC ACC ATT GTG ATT TTT TTA GCT GCT TTG CTG |
| EW7 | GCA GGC ATG CAA GCT TTC GAT AAG GCT TTC TGA AGG GGT GAT C |
| EW209 | TAA CGG AAA GTT TTT ACC GCG CAG ACA ATG AAT TTC CCG C |
| EW210 | AAA AAC TTT CCG TTA CAG CAC TGG CTG CGC |
| EW213 | ACC ACG CCT GAC AGA CTA AGT AAG ATG GGG AAA GCA TGA GCA CCA TTG TGA TTT TTT TAG CTG CTT TGC |
| EW214 | GAC AGG GTA GCA TAA CCT GCC GCG CAA ACG TGT TAT TCG ATA AGG CTT TCT GAA GGG GTG ATC AGT TG |
| Δ*atoS-rcsC154.CmF* | GCGTTCCACTGGCATATCACGCAGACCGAAATTGGCCATAAAATGAGACGTTGATCGGCACG |
| Δ*atoS-rcsC154.CmR* | GGTTAAGGTGATGATTTCTCGGCGGTGTATCATATTCCAGACCAGCAATAGACATAAGCGGGC |
| Cys111-ala_cat | CAGGCGGATGTGCCTGCGTTTGAACCGCTGTTTGCCGACTCCGATGCAAAATGAGCAGTTAGTCGGCAC |
| Cys111-ala_sacB | CGCCAATGACTCCAGAGAACCTCGCCAGGTGTTACTCATGTAGACTGCAAAGGGAAAACTGTCCATATG |
| Cys111Ala replacement primer | CAGGCGGATGTGCCTGCGTTTGAACCGCTGTTTGCCGACTCCGATGCTTCCGCAATGAGTAACACCTGGCGAGGTTCTCTGGAGTCATTGGCG |
| RcsC-KAN-AtoS.F | GGTAGCGGTAAAAGCGTGTTACCGCAATGTTCTCTCTTCTGTGTAGGCTGGAGCTGCTTCG |
| RcsC-KAN-AtoS.R | GGTTAAGGTGATGATTTCTCGGCGGTGTATCATATTCCAGATTCCGGGGATCCGTCGACC |
| atoS_RcsCys154 | CGCGTTCCACTGGCATATCACGCAGACCGAAATTGGCCATAGCGAGGTTATCGCTGCCGATTAAAAATAC |
| cpsB-zeo.R | ATT TAC ACC GCG GTT TCG CAT TCA TTG CCT GAT GCG ACG TAA AAA AAG CCC GCT CAT TAG |
| wza-zeo.F | GTG CAC AGG ATA ATT ACT CTG CCA AAG TGA TAA ATA AAC AGT TGA CAA TTA ATC ATC GGC |
| yrfF_pBAD24F | CAG GAG GAA TTC ATG AGC ACC ATT GTG ATT TTT TTA GC |
| yrfF_pBAD24R | ACA GCC AAG CTT TTA TTC GAT AAG GCT TTC TG |
| yrfF Cys425Ser.F | TTT TTA CCG TTT GAC AGC TCG CAG ATC ATC T |
| yrfF Cys425Ser.R | AGA TGA TCT GCG AGC TGT CAA ACG GTA AAA A |
| yrfF Cys498-504Ser.F | AAG ACA GCG GAT TTA AGT TCT GCC AAA GAT GAC TGA GTG CGA CTG AAA AAT |
| yrfF Cys498-504Ser.R | ATT TTT CAG TCG CAC TCA GTC ATC TTT GGC AGA ACT TAA ATC CGC TGT CTT |
| yrfF Cys404Ser.F | AGC GGT ACG GGA ATG AGT AAT ATT CGA ACT T |
| yrfF Cys404Ser.R | AAG TTC GAA TAT TAC TCA TTC CCG TAC CGC T |
| AP241 | GCT ATG ACC ATG ATT GAA TAC GGT TCG ACA AAG ATG GAA GAG AGA CTC T |
| AP242 | GCA GGC ATG CAA GCT TTC ATA GCG TCT GCT ACG TGC GG |
| AP321 | GGG AAT CCG GCT CAT CAC CCA GAT GTA AC |
| AP322 | ATG AGC CGG ATT CCC TCT ACC GCG GGA AAG TGG TTA GGG |
| AP323 | CAT TGC CCA GAC GAA ATA GCC CGC CCA TGT ATA GAA AGC CCA CAA |
| AP324 | TTG TGG GCT TTC TAT ACA TGG GCG GGC TAT TTC GTC TGG GCA ATG |
| AP325 | CTC ATC ACC CAG ATG TAA AAC GCC ATT GCC CAG ACG AA |
| AP326 | TTC GTC TGG GCA ATG GCG TTT TAC ATC TGG GTG ATG AG |
| AP327 | ACC AGG CAA TGC TGG CTA ACA ATG CCC CG |
| AP328 | CGG GGC ATT GTT AGC CAG CAT TGC CTG GT |
| AP329 | GGA CGA GGT CGG GCG TAC CAG GCA ATG |
| AP330 | CAT TGC CTG GTA CGC CCG ACC TCG TCC |
| AP351 | CGG AAG TTC GAA TAT TAG ACA TTC CCG TAC CGC TA |
| AP352 | TAG CGG TAC GGG AAT GTC TAA TAT TCG AAC TTC CG |
| AP375 | TTA TAT CGA TTG GCG TTC CAC TGC G |
| AP376 | CTA AGT AAT ATG GTG CAC TCT CAG TAC AAT CTG CTC |
| AP459 | CAGCTTATCGGGCTGGTGTTCACTCATCAGCTTACG |
| AP460 | CGTAAGCTGATGAGTGAACACCAGCCCGATAAGCTG |
| AP495 | CCC TGG ACC AAC GCC TTT CCG TTA CAG CAC TGG CTG CGC AGT AC |
| AP496 | ATG TTT GAT AAA GCC CGT AGC CGT AAA ATG G |
| AP501 | CGA CAG GGG ATC TGC TTA ACT GGT CAC AGC CGC TTA GCA TGA GTG |
| AP502 | CAC TCA TGC TAA GCG GCT GTG ACC AGT TAA GCA GAT CCC CTG TCG |
| AP559 | GAG CTC GAA TTC GCT AGC CCA AAA AAA CG |
| AP560 | AAG CTT GGC TGT TTT GGC GGA TGA G |
| AP563 | GGC TTT ATC AAA CAT ATA TTC CCC AGA TCG ACA CAC GGA TG |
| AP564 | ATG TTT GAT AAA GCC CGT AGC CGT AAA ATG G |
| AP565 | AGC GAA TTC GAG CTC AGC AGG AGG AAT TCA ATG GAT GTC ATT AAA AAG AAA C |
| AP566 | AAA ACA GCC AAG CTT TCA TTT AAA CCC TTT CTG CTG CTT TAT CAG |
| AP587 | CAT TTT TCA GTC GCA CAC TGT CAT CTT TGG CAG AAC A |
| AP588 | TGT TCT GCC AAA GAT GAC AGT GTG CGA CTG AAA AAT G |
| AP691 | CCA GAT GAT CTG CGA GCT GTC AAA CGG TAA AAA AG |
| AP692 | CTT TTT TAC CGT TTG ACA GCT CGC AGA TCA TCT GG |
| AP693 | CAT CTT TGG CAG AAG ATA AAT CCG CTG TCT TCA GTA C |
| AP694 | GTA CTG AAG ACA GCG GAT TTA TCT TCT GCC AAA GAT G |
