# Supplementary material for: RcsF-independent mechanisms of signaling within the Rcs phosphorelay
Source: PLoS Genet. 2024 Dec 26;20(12):e1011408. doi: 10.1371/journal.pgen.1011408 (PMC11709261; doi:10.1371/journal.pgen.1011408)
Supplement: S4 Table — (DOCX) [file pgen.1011408.s004.docx]

**TableS4: List of gBlocks used in this study**

| **gBlock name** | **Sequence** |
| --- | --- |
| **AP_GymgB** | AGCGAATTCGAGCTCAGCAGGAGGAATTCAATGCTTGAAGATACTACAATTCATAATGCAATAACTGATAAAGCGTTAGCAAGTTACTTTCGCAGTTCGGGTAATTTGTTAGAAGAAGAATCAGCAGTGTTAGGGCAGGCTGTCACCAATTTAATGCTTTCAGGCGATAATGTTAATAATAAAAATATTATCTTAAGTCTGATACACTCCTTGGAAACAACAAGTGATATTCTCAAAGCTGATGTGATTAGAAAAACACTGGAAATCGTGTTGCGATACACAGCTGATGATATGTAAGCTTGGCTGTTTT |
| **AP_GyihA** | AGCGAATTCGAGCTCAGCAGGAGGAATTCAATGACTAATTTGAATTATCAACAGACGCATTTTGTGATGAGTGCGCCTGATATTCGCCACCTACCTTCCGATACCGGAATTGAAGTGGCTTTTGCAGGCCGTTCCAACGCAGGTAAATCCAGCGCGCTGAACACGCTGACTAACCAGAAAAGCCTGGCTCGTACCTCAAAAACCCCAGGGCGCACCCAGCTTATCAACCTGTTTGAAGTGGCTGACGGCAAGCGTCTGGTTGACTTGCCTGGGTACGGTTATGCGGAAGTCCCGGAAGAGATGAAGCGCAAATGGCAGCGTGCGCTCGGCGAATACCTCGAAAAACGTCAGAGCCTGCAAGGTCTGGTGGTGCTAATGGATATTCGCCATCCGCTGAAAGATTTGGATCAGCAGATGATTGAGTGGGCGGTAGACAGCAATATCGCCGTTCTGGTGCTGCTGACCAAAGCGGACAAACTGGCAAGCGGCGCACGTAAAGCGCAATTGAATATGGTGCGTGAAGCTGTACTGGCGTTTAACGGTGATGTGCAGGTTGAAACGTTTTCTTCGTTGAAGAAACAAGGCGTGGACAAGCTGCGGCAGAAACTGGATACCTGGTTTAGCGAGATGCAGCCTGTAGAAGAAACGCAGGACGGCGAATAAAAGCTTGGCTGTTTT |
| **AP_GF33** | AGCGAATTCGAGCTCAGCAGGAGGAATTCAATGCGTGCTTTACCGATCTGTTTAGTAGCACTCATGCTAAGCGGCTGTTCCATGTTAAGCAGATCCCCTGTCGAACCCGTTCAAAGCACTGCACCCCAGCCGAAAGCGGAGCCTGCAAAACCGAAAGCGCCGCGCGCCACGCCGGTCCGAATTTATACCAATGCAGAAGAATTAGTCGGCAAACCGTTCCGCGATCTCGGTGAAGTCAGTGGCGACTCTTGCCAGGCCTCTAATCAGGACTCTCCGCCGAGCATTCCAACCGCACGTAAGCGGATGCAAATCAACGCCTCTAAAATGAAAGCCAATGCTGTATTACTGCATAGCTGCGAAGTCACCAGCGGTACGCCAGGCTGCTATCGTCAGGCTGTATGTATCGGTTCTGCGCTTAACATTACGGCGAAATGAAAGCTTGGCTGTTTT |
| **AP_GJ1** | CGCCAATCGATATAAAGCAGGAGGAATTCAATGCAGTATTGGGGAAAAATCATTGGCGTGGCCGTGGCCTTACTGATGGGCGGCGGCTTTTGGGGCGTAGTGTTAGGCCTGTTAATTGGCCATATGTTTGATAAAGCCCGTAGCCGTAAAATGGCGTGGTTCGCCAACCAGCGTGAGCGTCAGGCGCTGTTTTTTGCCACCACTTTTGAAGTGATGGGGCATTTAACCAAATCCAAAGGTCGCGTCACGGAGGCTGATATTCATATCGCCAGCCAGTTGATGGACCGAATGAATCTTCATGGCGCTTCCCGTACTGCGGCGCAAAATGCGTTCCGGGTGGGAAAATCAGACAATTACCCGCTGCGCGAAAAGATGCGCCAGTTTCGCAGTGTCTGCTTTGGTCGTTTTGACTTAATTCGTATGTTTCTGGAGATCCAGATTCAGGCGGCGTTTGCTGATGGTTCACTGCACCCGAATGAACGGGCGGTGCTGTATGTCATTGCAGAAGAATTAGGGATCTCCCGCGCTCAGTTTGACCAGTTTTTGCGCATGATGCAGGGCGGTGCACAGTTTGGCGGCGGTTATCAGCAGCAAACTGGCGGTGGTAACTGGCAGCAAGCGCAGCGTGGCCCAACGCTGGAAGATGCCTGTAATGTGCTGGGCGTGAAGCCGACGGATGATGCGACCACCATCAAACGTGCCTACCGTAAGCTGATGAGTGAACACCATCCCGATAAGCTGGTGGCGAAAGGTTTGCCGCCTGAGATGATGGAGATGGCGAAGCAGAAAGCGCAGGAAATTCAGCAGGCATATGAGCTGATAAAGCAGCAGAAAGGGTTTAAATGACTAAGTAATATGGTG |
| **AP_GJMF** | CGCCAATCGATATAAAGCAGGAGGAATTCAATGGATGTCATTAAAAAGAAACATTGGTGGCAAAGCGACGCGCTGAAATGGTCAGTGCTAGGTCTGCTCGGCCTGCTGGTGGGTTACCTTGTTGTTTTAATGTACGCACAAGGGGAATACCTGTTCGCCATTACCACGCTGATATTGAGTTCAGCGGGGCTGTATATGTTTGATAAAGCCCGTAGCCGTAAAATGGCGTGGTTCGCCAACCAGCGTGAGCGTCAGGCGCTGTTTTTTGCCACCACTTTTGAAGTGATGGGGCATTTAACCAAATCCAAAGGTCGCGTCACGGAGGCTGATATTCATATCGCCAGCCAGTTGATGGACCGAATGAATCTTCATGGCGCTTCCCGTACTGCGGCGCAAAATGCGTTCCGGGTGGGAAAATCAGACAATTACCCGCTGCGCGAAAAGATGCGCCAGTTTCGCAGTGTCTGCTTTGGTCGTTTTGACTTAATTCGTATGTTTCTGGAGATCCAGATTCAGGCGGCGTTTGCTGATGGTTCACTGCACCCGAATGAACGGGCGGTGCTGTATGTCATTGCAGAAGAATTAGGGATCTCCCGCGCTCAGTTTGACCAGTTTTTGCGCATGATGCAGGGCGGTGCACAGTTTGGCGGCGGTTATCAGCAGCAAACTGGCGGTGGTAACTGGCAGCAAGCGCAGCGTGGCCCAACGCTGGAAGATGCCTGTAATGTGCTGGGCGTGAAGCCGACGGATGATGCGACCACCATCAAACGTGCCTACCGTAAGCTGATGAGTGAACACCATCCCGATAAGCTGGTGGCGAAAGGTTTGCCGCCTGAGATGATGGAGATGGCGAAGCAGAAAGCGCAGGAAATTCAGCAGGCATATGAGCTGATAAAGCAGCAGAAAGGGTTTAAATGACTAAGTAATATGGTG |
