# Supplementary figures and images for: RcsF-independent mechanisms of signaling within the Rcs phosphorelay
Source: PLoS Genet. 2024 Dec 26;20(12):e1011408. doi: 10.1371/journal.pgen.1011408 (PMC11709261; doi:10.1371/journal.pgen.1011408)

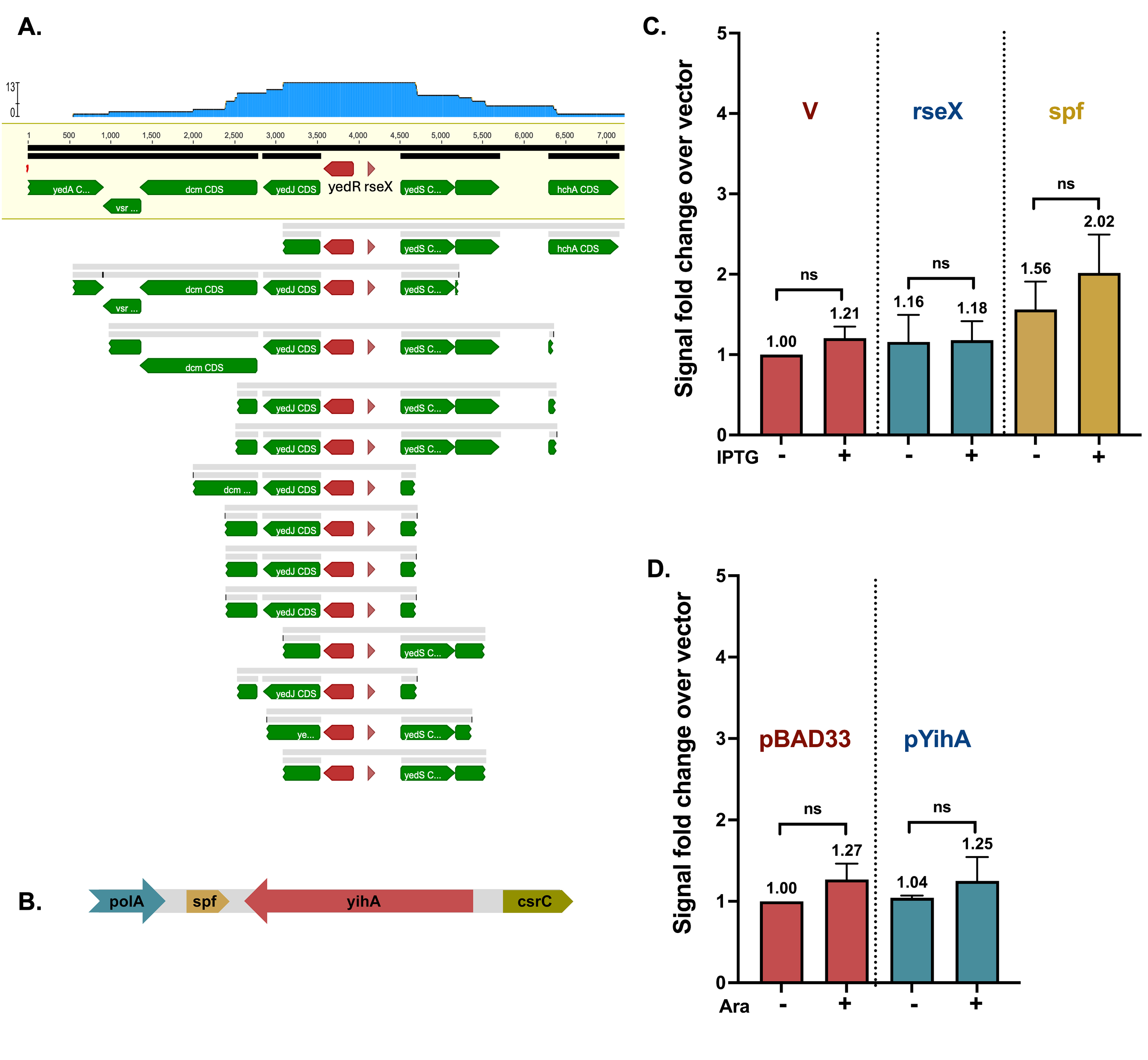

Supplement: S1 Fig — A genomic library was transformed into an rcsF deletion strain (EAW34) containing the PrprA::mCherry reporter for Rcs signaling and the colonies were screened for increased fluorescence. The plasmids were isolated from colonies showing high fluorescence (indicating Rcs activation) and sequenced. The genes present in the candidate plasmids, representing two genomic regions, are shown here. A. The gene encoding the YedR/DrpB ORF (indicated here in red) was present in 13 out of 14 candidate plasmids, in 8 independent clones with different end points. B. The remaining candidate plasmid contained genes for Spot42 sRNA (spf) and the YihA ORF. C. For this PrprA::mCherry assay, the strain AP 51 (rcsF::kan) overexpressing pBR-plac (V), pBR-plac-rseX or pBR-plac-spf was grown in MOPS minimal glycerol medium containing 100 μg/ml ampicillin and their fluorescence measured over time at 37°C. The RFU at OD 0.4 is plotted with the value for the vector in the absence of IPTG set to 1. The strains were induced with 100 μM IPTG. D. The AP51 strain containing pBAD33 or pBAD33-yihA (pAP3327) was grown in MOPS minimal glycerol medium with 25 μg/ml chloramphenicol and induced with 0.02% arabinose. Data from three independent experiments is plotted as mean with error bars indicating the standard deviation. Values were statistically analyzed using multiple unpaired t-tests. ‘ns’ indicates a P-value > 0.05 (non- significant). (TIF) [file pgen.1011408.s006.tif]

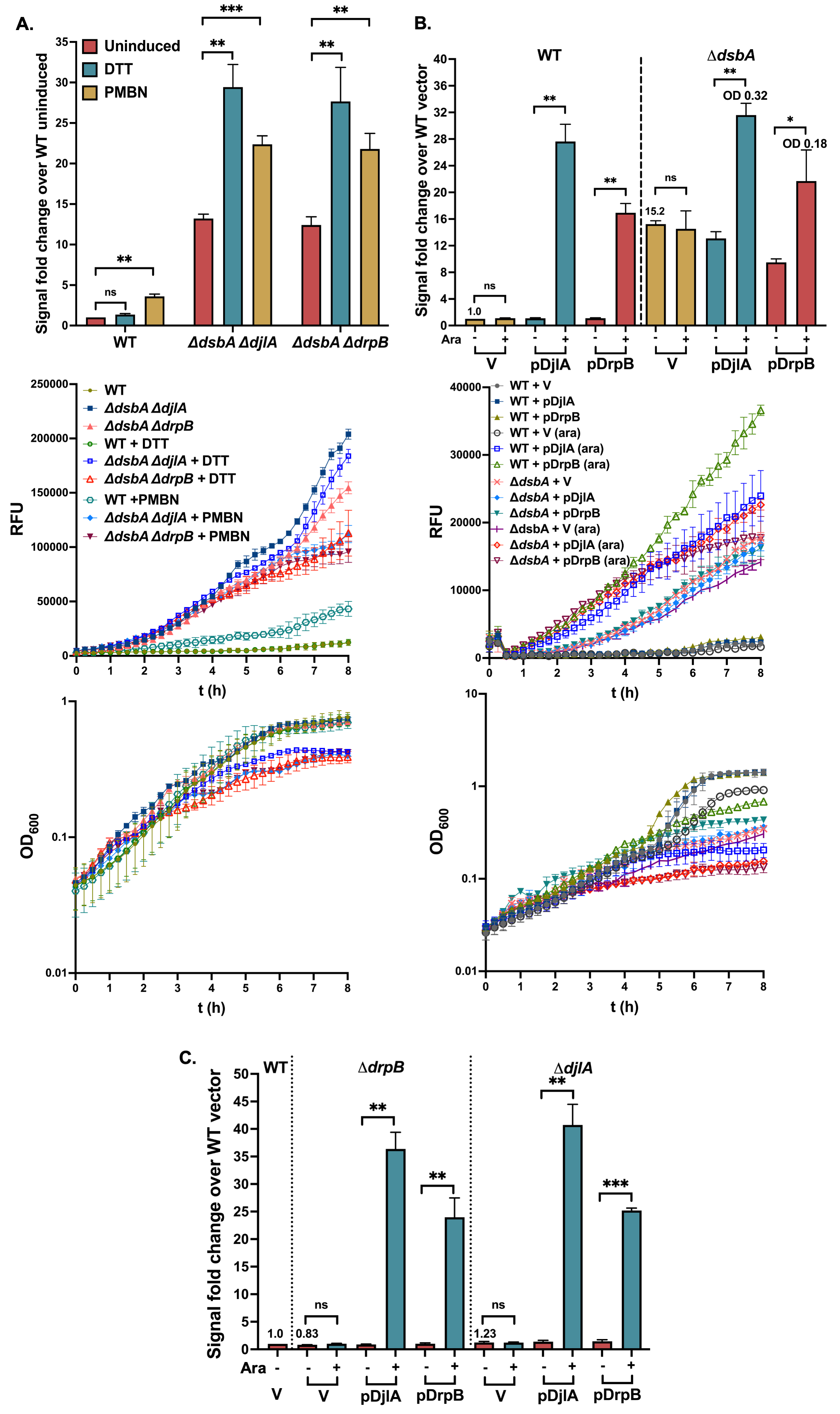

Supplement: S2 Fig — A. Effect of DjlA or DrpB deletion upon Rcs signaling in dsbA mutants: All the strains carry an rprA promoter fusion to mCherry (PrprA-mCherry) and the mCherry fluorescence acts as an indicator for Rcs activation. For the PrprA::mCherry assay, the cells were grown in MOPS minimal glucose medium at 37°C. The RFU at OD 0.4 for the WT uninduced was set to 1 and the relative induction compared to that depicted here (top panel). The cells were treated at the beginning of growth with either 1mM DTT or 20μg/ml PMBN. Strains used were: WT (EAW8), ΔdsbA djlA::kan (AP72), and ΔdsbA drpB::kan (AP71). The middle panel depicts the relative fluorescence units (RFU) as a function of time and the lower panel shows a representative growth curve for each strain. B. Signaling upon overexpression of DjlA or DrpB in dsbA mutants: The strains WT (EAW8) and dsbA::kan (EAW62) carry the PrprA::mCherry promoter fusion. The strains containing plasmids overexpressing DjlA (pDjlA/pPSG961) or DrpB (pDrpB/pDSW1977) were grown in MOPS minimal glycerol medium with 25 μg/ml chloramphenicol at 37°C and 0.02% arabinose was added for induction. The RFU at OD 0.4 for the WT vector uninduced was set to one; other results are normalized to that value. The middle and bottom panels depict the RFU and OD600 as a function of time. C. Rcs activation by overexpression of DjlA and DrpB in ΔdrpB and ΔdjlA strains: The strains overexpressing DjlA (pDjlA/pPSG961) or DrpB (pDrpB/pDSW1977) were grown as in B. Strains used were: WT (EAW8), drpB::kan (AP41), and djlA::kan (AP46). Data from three independent experiments is plotted as mean with error bars indicating the standard deviation. Values were statistically analyzed using multiple unpaired t-tests. Statistical significance is indicated as follows: ns (P > 0.05; non- significant), * (P < 0.05), ** (P ≤ 0.01), *** (P ≤ 0.001). (TIF) [file pgen.1011408.s007.tif]

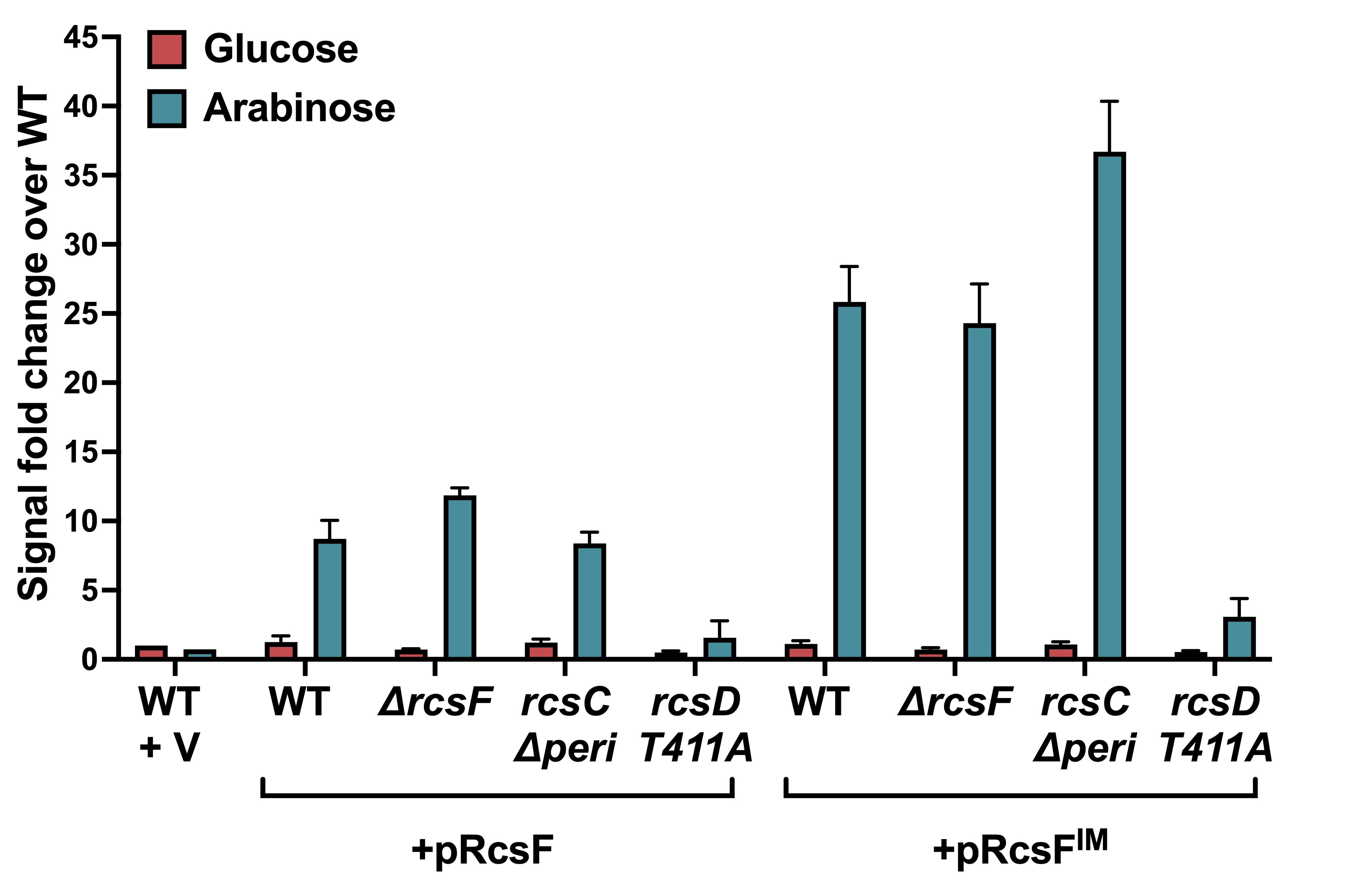

Supplement: S3 Fig — The strains overexpressing RcsF (pAP3340) or its inner membrane localized mutant RcsFIM (pAP3341) were grown in MOPS minimal glycerol medium containing chloramphenicol (25 μg/ml) and either 0.2% glucose or 0.02% arabinose at 37°C. The RFU at OD 0.4 compared to the uninduced vector control, set to 1, was plotted. The strains used were: WT (EAW8), rcsF::kan (AP51), rcsCΔperi (EAW70), and rcsD T411A (EAW121). (TIF) [file pgen.1011408.s008.tif]

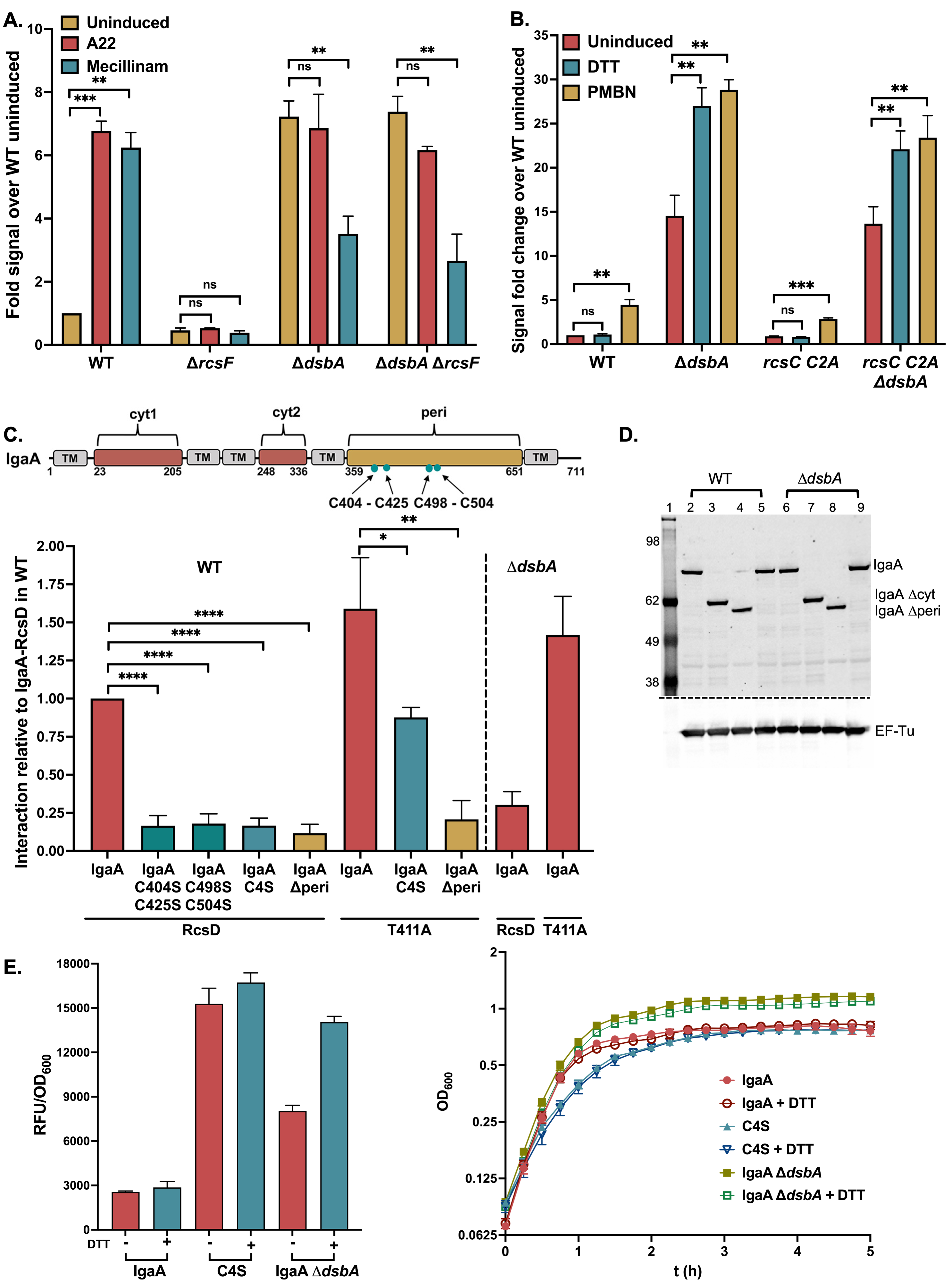

Supplement: S4 Fig — For the PrprA::mCherry assay, the strains were grown in MOPS minimal glucose medium at 37°C. The RFU at OD 0.4 as compared to WT uninduced, set to 1, is depicted here. The cells were subjected to either 5 μg/ml A22 or 0.3 μg/ml mecillinam. The strains used were: WT (EAW8), rcsF::cat (EAW32), dsbA::kan (EAW62), and dsbA::kan rcsF::cat (EAW67). B. Rcs signaling in RcsC cysteine mutant strains. For the PrprA::mCherry assay, the strains were grown in MOPS minimal glucose medium at 37°C. The RFU at OD 0.4 as compared to WT uninduced, set to one, is shown here. The cells were subjected to either 1mM DTT or 20μg/ml PMBN. The dsbA RcsC C2A (rcsC C111A C154A) mutant responded to DTT and PMBN similarly to the ΔdsbA strain. The strains used were: WT (EAW8), dsbA::kan (EAW62), rcsC C2A atoS::kan (AP172), and ΔdsbA rcsC C2A atoS::kan (AP173). C. Interaction of IgaA Cysteine mutants with RcsD. Beta-galactosidase activity was measured in bacterial two hybrid assay, in the standard cyaA mutant strain (BTH101 or in a strain mutant for both cyaA and dsbA, AP 58 (BTH101 ΔdsbA)). The IgaA-RcsD interaction in WT was normalized to 1 and other interactions plotted relative to this interaction. The interaction of IgaA-RcsD was 569 units, while the vector control was 20; these units are 1000x the slope of OD420 (see Materials and Methods). This data is compiled from separate sets of assays, each normalized relative to the IgaA/RcsD signal in that experiment. The T18 tagged plasmids used are pEAW1 (IgaA-T18), pAP102 (IgaA C404S C425S), pAP103 (IgaA C498S C504S), pEAW1C4S (IgaA C4S) pEAW1peri (IgaA Δperi), pEAW8 (RcsD-T25), and pEAW8T (RcsD T411A-T25). See Fig 4B for IgaAΔperi structure. D. Expression of IgaA-T18 fusions. EAW8 (WT) and EAW62 (ΔdsbA) expressing the IgaA-T18 fusion constructs were probed with the anti-CyaA antibody and anti-EF-Tu antibody (loading control). Lane 1 shows the protein markers; Lanes 2–5 and Lanes 6–9 show expression in EAW8 and EAW62 respectively. Lanes 2, 6: pEAW [file pgen.1011408.s009.tif]

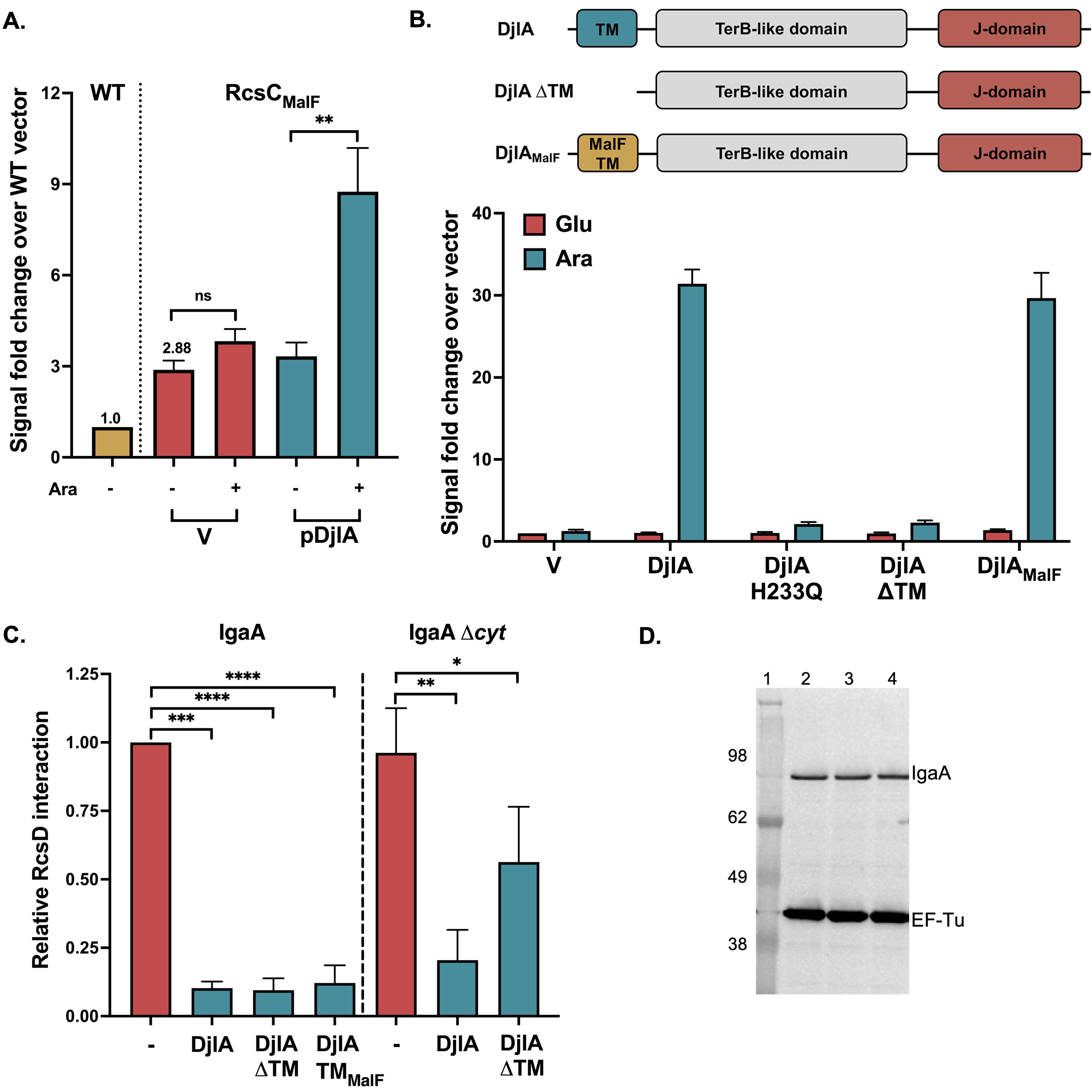

Supplement: S5 Fig — A. DjlA signaling in a RcsCMalF strain: For the PrprA::mCherry assay, the strains WT (EAW8) and RcsCMalF (EAW72) overexpressing DjlA (pDjlA/pPSG961) or pBAD33 vector were grown in MOPS minimal glycerol medium containing chloramphenicol (25 μg/ml) and either 0.2% glucose or 0.02% arabinose at 37°C. The RFU at OD 0.4 uninduced for the WT was set to 1 (first column), and relative values to that are plotted. EAW72 carries a chimeric variant of RcsC which has its cytoplasmic domains fused to the MalF TM helices. Statistical significance was calculated using multiple unpaired t-tests and is indicated as follows: ns (P > 0.05; non- significant), * (P < 0.05), ** (P ≤ 0.01), *** (P ≤ 0.001), and **** (P ≤ 0.0001). B. Signaling by DjlA mutants: For the PrprA::mCherry assay, the strain AP 163 (djlA::kan ΔrcsF) carrying the pBAD33 vector or pBAD33 derivatives expressing DjlA (pDjlA/pPSG961), DjlA H233Q (pAP3315), DjlA ΔTM (pAP3311), or DjlAMalF (pAP3312) were grown as in A. The RFU of the strain carrying the uninduced vector at OD 0.4 was set to 1 and results normalized to that are plotted. C. The DjlA TM helix is not needed for weakening IgaA-RcsD interactions. Beta-galactosidase activity was measured in a cyaA mutant strain (BTH101). IgaA and RcsD/RcsD T411A were fused to the T18 and T25 domains, respectively. DjlA or its variants were cloned downstream of IgaA under the same promoter control. IgaA-RcsD interaction in the absence of DjlA was normalized to 1 and all other interactions are plotted relative to this interaction. The interaction of IgaA-RcsD was 580 units, while the vector control was 27; these units are 1000x the slope of OD420 (see Materials and Methods). Plasmids used were pEAW1 (IgaA-T18), pEAW8 (RcsD-T25), pAP101 (IgaA Δcyt–T18), pAP1401 (IgaA-T18 + DjlA), pAP1403 (IgaA-T18 + DjlA ΔTM), and pAP1404 (IgaA-T18 + DjlAMalF). D. Expression of IgaA-T18 fusion. E. coli DH5α strain expressing the IgaA-T18 fusion constructs were probed with the anti-CyaA antibody and [file pgen.1011408.s010.tif]

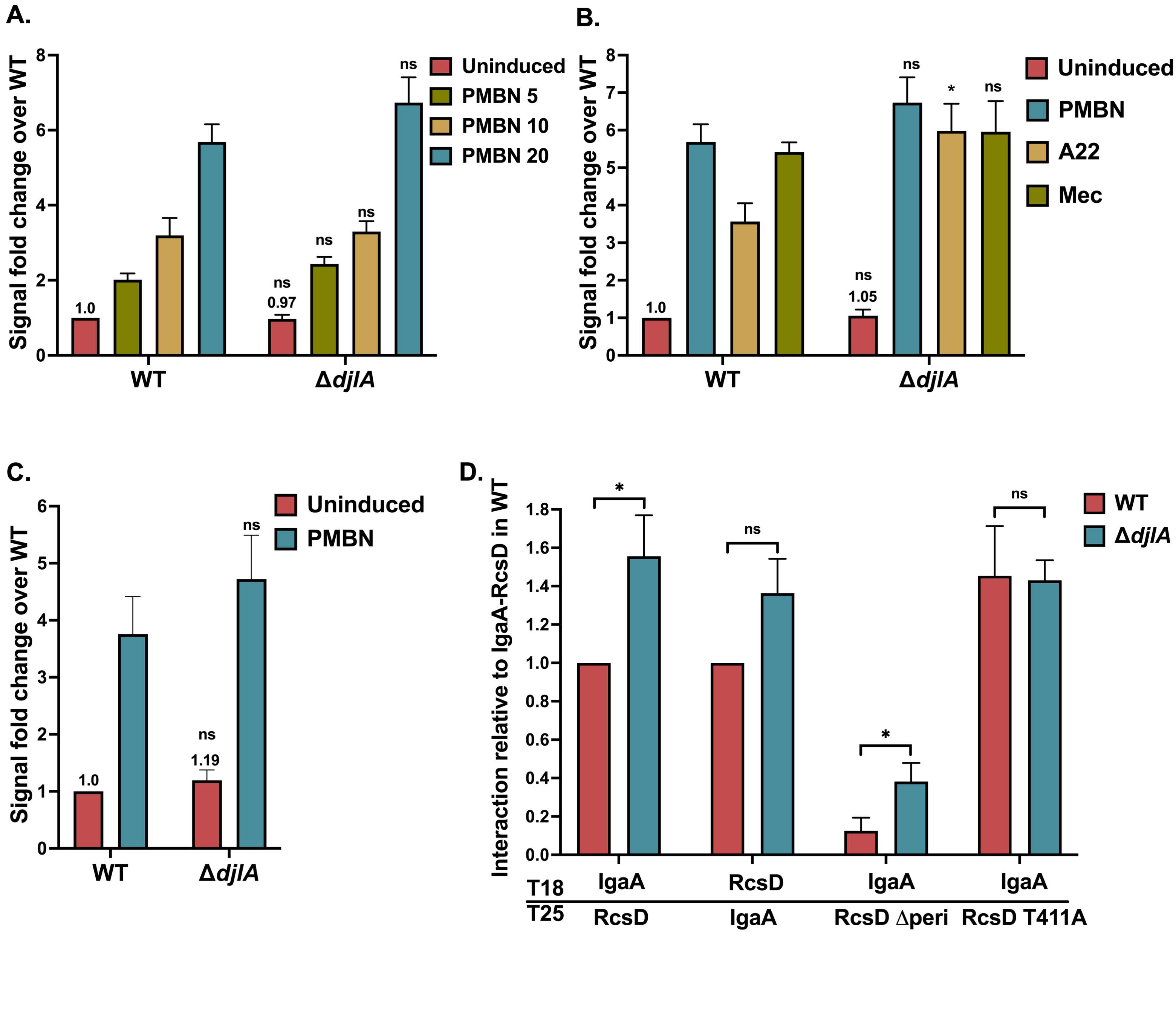

Supplement: S6 Fig — A. Dose-dependent PMBN response in ΔdjlA: For the PrprA::mCherry assay, the strains WT (EAW8) and djlA::kan (AP46) were grown in MOPS minimal glucose medium with or without PMBN (5 μg/ml, 10 μg/ml, or 20 μg/ml) at 37°C. The RFU at OD 0.4 as compared to WT uninduced, set to 1, is depicted here. Values (Mean ± SD) from independent experiments were statistically analyzed using multiple unpaired t-tests. The fluorescence signal of each PMBN dose in ΔdjlA was compared to the RFU signal of the WT strain at the same dose. Statistical significance for this comparison is indicated as follows: ns (P > 0.05; non- significant), * (P ≤ 0.05). B. ΔdjlA responds to A22 and Mecillinam: For the PrprA::mCherry assay, the strains WT (EAW8) and djlA::kan (AP46) were grown in MOPS minimal glucose medium and treated with nothing (uninduced) or with PMBN (20 μg/ml), MreB inhibitor A22 (5 μg/ml), or Mecillinam (0.3 μg/ml, Mec) at 37°C. The RFU at OD 0.4 as compared to WT uninduced, set to 1, is plotted. For statistical analysis, the RFU with each chemical treatment in ΔdjlA was compared to the RFU signal of the WT strain for that compound. C. PMBN response in ΔdjlA at 42°C: For the PrprA::mCherry assay, the strains WT (EAW8) and djlA::kan (AP46) were grown in MOPS minimal glucose medium with or without PMBN (20 μg/ml) at 42°C. The RFU at OD 0.4 as compared to WT uninduced is plotted. The statistical analysis shows the comparison between signals of ΔdjlA and WT. D. IgaA-RcsD interactions in ΔdjlA: The IgaA-RcsD interaction was tested using BACTH in BTH101 (WT) and AP 63 (BTH 101 ΔdjlA). Plasmids used were pEAW1 (IgaA-T18), pEAW8 (RcsD-T25), pEAW2 (IgaA-T25), pEAW7 (RcsD-T18), pEAW8peri (RcsD Δperi-T25), and pEAW8T (T411A). IgaA-RcsD interaction in WT was normalized to 1 and the interaction in ΔdjlA (AP63) is plotted relative to this interaction. The interaction of IgaA-RcsD was 578 units, while the vector control was 25; these units are 1000x the slope of OD420 (see Materials and Methods). [file pgen.1011408.s011.tif]

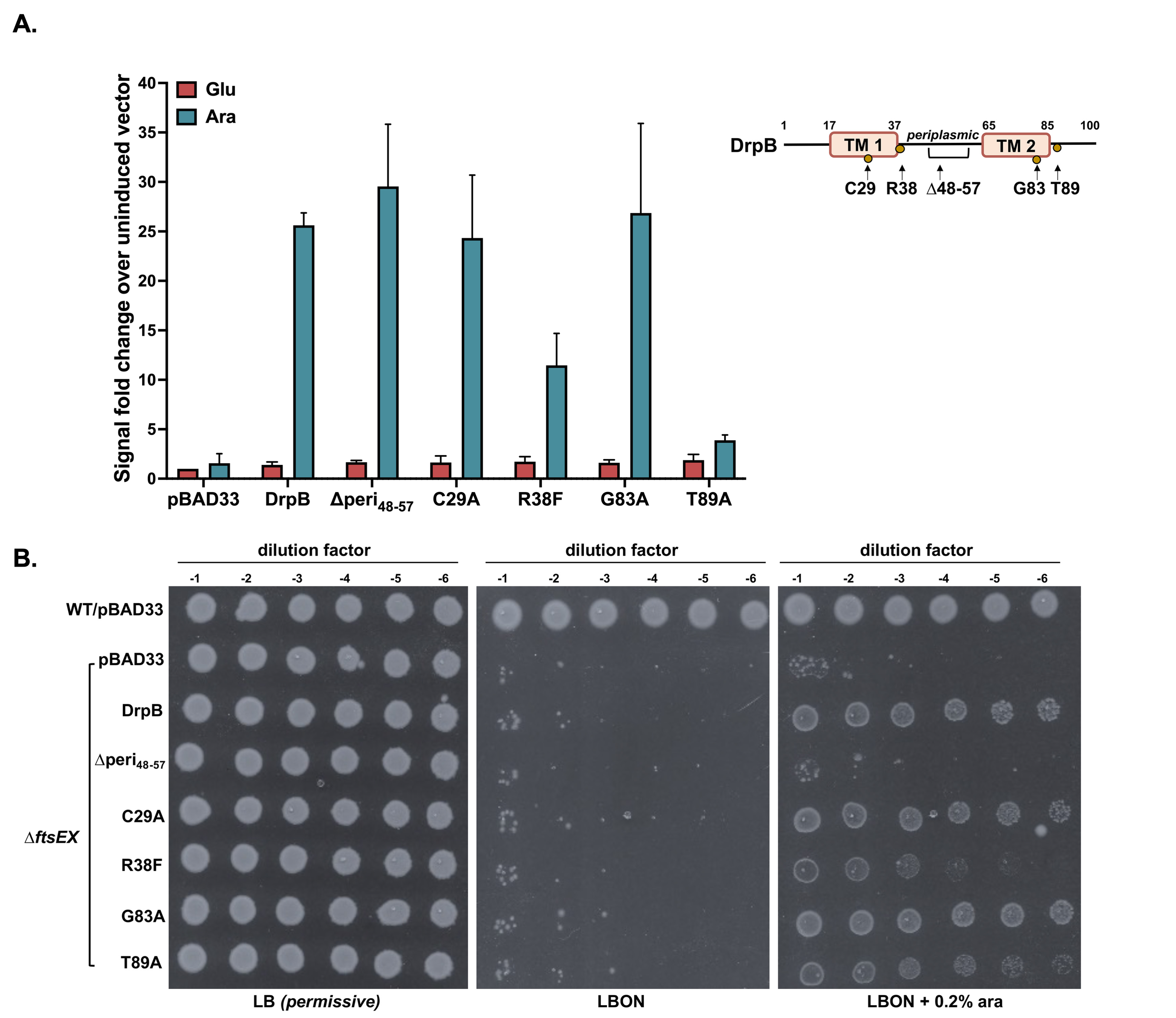

Supplement: S7 Fig — A. Signaling by DrpB mutants in ΔrcsF: For the PrprA::mCherry assay, the rcsF::kan (AP51) strain was transformed with the pBAD33 vector or pBAD33 plasmids carrying DrpB (pDSW1977), DrpB Δperi48-57 (pAP3301), DrpB C29A (pAP3304), DrpB R38F (pAP3305), DrpB G83A (pAP3306), or DrpB T89A (pAP3307). The transformed strains were grown in MOPS minimal glycerol medium containing chloramphenicol (25 μg/ml) and either 0.2% glucose or 0.02% arabinose at 37°C. The RFU at OD 0.4 is plotted relative to the uninduced WT, set to 1. B. Analysis of DrpB mutants as ftsEX suppressors: The strains WT (EC251) and ΔftsEX (EC1215) were transformed with the same plasmids as in A and grown overnight in LB Miller media with chloramphenicol at 37°C. The cultures were then normalized to OD600 1 and 4 ul dilutions were spotted on LB Miller (permissive), LBON (LB without NaCl) or LBON (with 0.2% arabinose). Plates were imaged after 16h incubation at 37°C. (TIF) [file pgen.1011408.s012.tif]

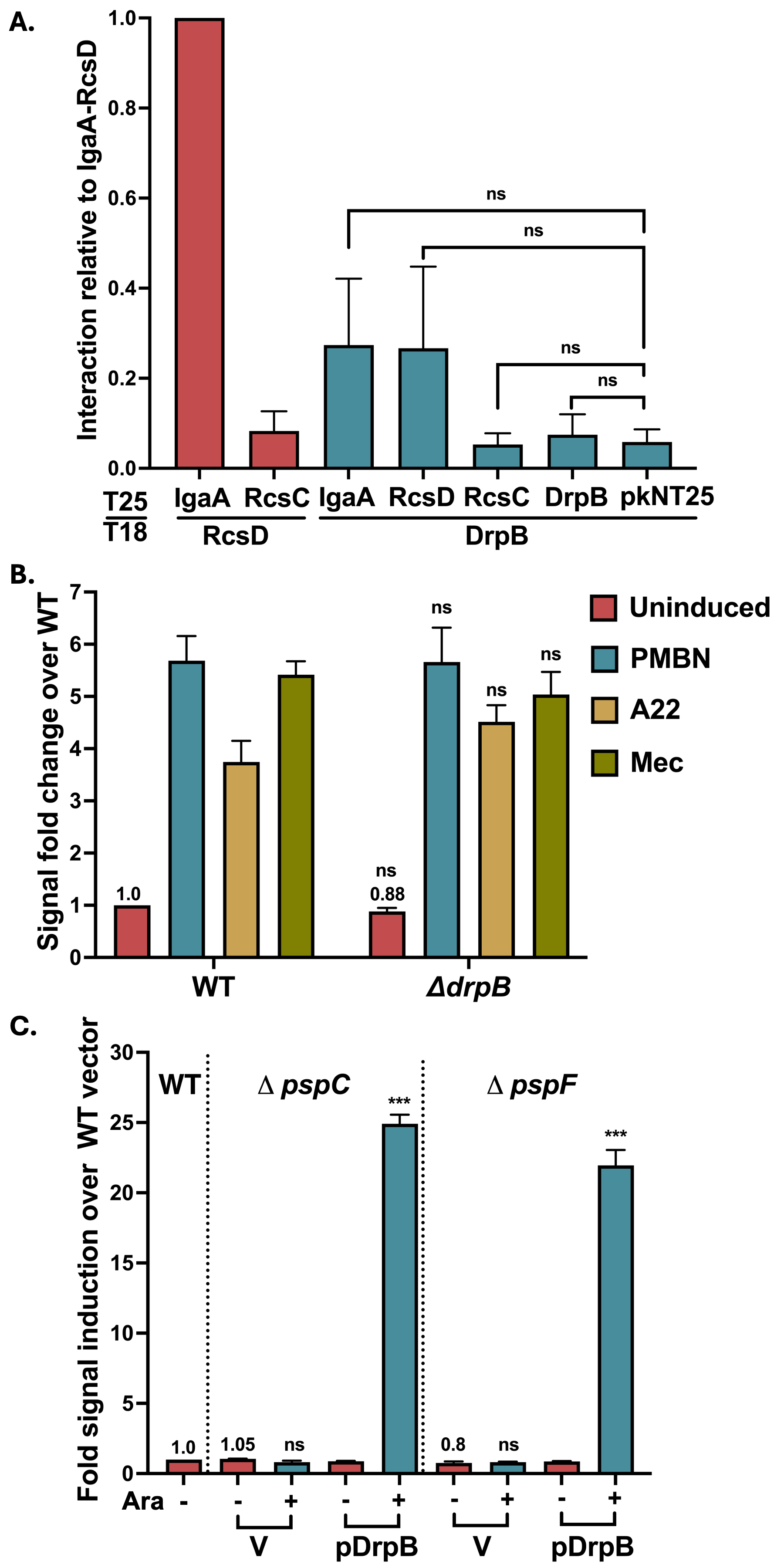

Supplement: S8 Fig — A. DrpB interactions with Rcs components: The interaction of DrpB with IgaA, RcsD, and RcsC was tested using the BACTH assay in BTH101. The IgaA-RcsD interaction was normalized to 1 and the other interactions were plotted relative to this interaction. The interaction of IgaA-RcsD was 490 units, while the vector control was 23; these units are 1000x the slope of OD420 (see Materials and Methods). No significant interaction of RcsC was observed with RcsD or DrpB. Plasmids used were pEAW2 (IgaA-T25), pEAW8 (RcsD-T25), pEAW6 (RcsC-T25), pEAW7 (RcsD-T18), pAP407 (DrpB-T18), pAP408 (DrpB-T25), and pKNT25 vector. Values (Mean ± SD) from independent experiments were statistically analyzed using multiple unpaired t-tests. ‘ns’ indicates a P-value > 0.05 (non- significant). B. Rcs signaling by ΔdrpB: For the PrprA::mCherry assay, the strains WT (EAW8) and drpB::kan (AP41) were grown in MOPS minimal glucose medium and treated with nothing (uninduced) or with PMBN (20 μg/ml), MreB inhibitor A22 (5 μg/ml), or Mecillinam (0.3 μg/ml, Mec) at 18°C. The RFU at OD 0.4 as compared to WT uninduced, set to 1, is plotted. The statistical analysis compared the signals of ΔdrpB and WT for each condition. C. DrpB signaling is independent of the psp pathway. For this PrprA::mCherry assay, the WT (EAW8), pspC::kan (AP113) and pspF::kan (AP114) strains transformed with pBAD33 vector or pDrpB (pDSW1977) were grown in MOPS minimal glycerol medium containing chloramphenicol (25 μg/ml) either 0.2% glucose or 0.02% arabinose at 37°C. The RFU at OD 0.4 is plotted relative to uninduced WT, set to 1. Statistical analysis was done using multiple unpaired t-tests and P-value for comparison with the uninduced vector control of each strain is indicated as follows: ns (P > 0.05; non- significant) and *** (P ≤ 0.001). (TIF) [file pgen.1011408.s013.tif]

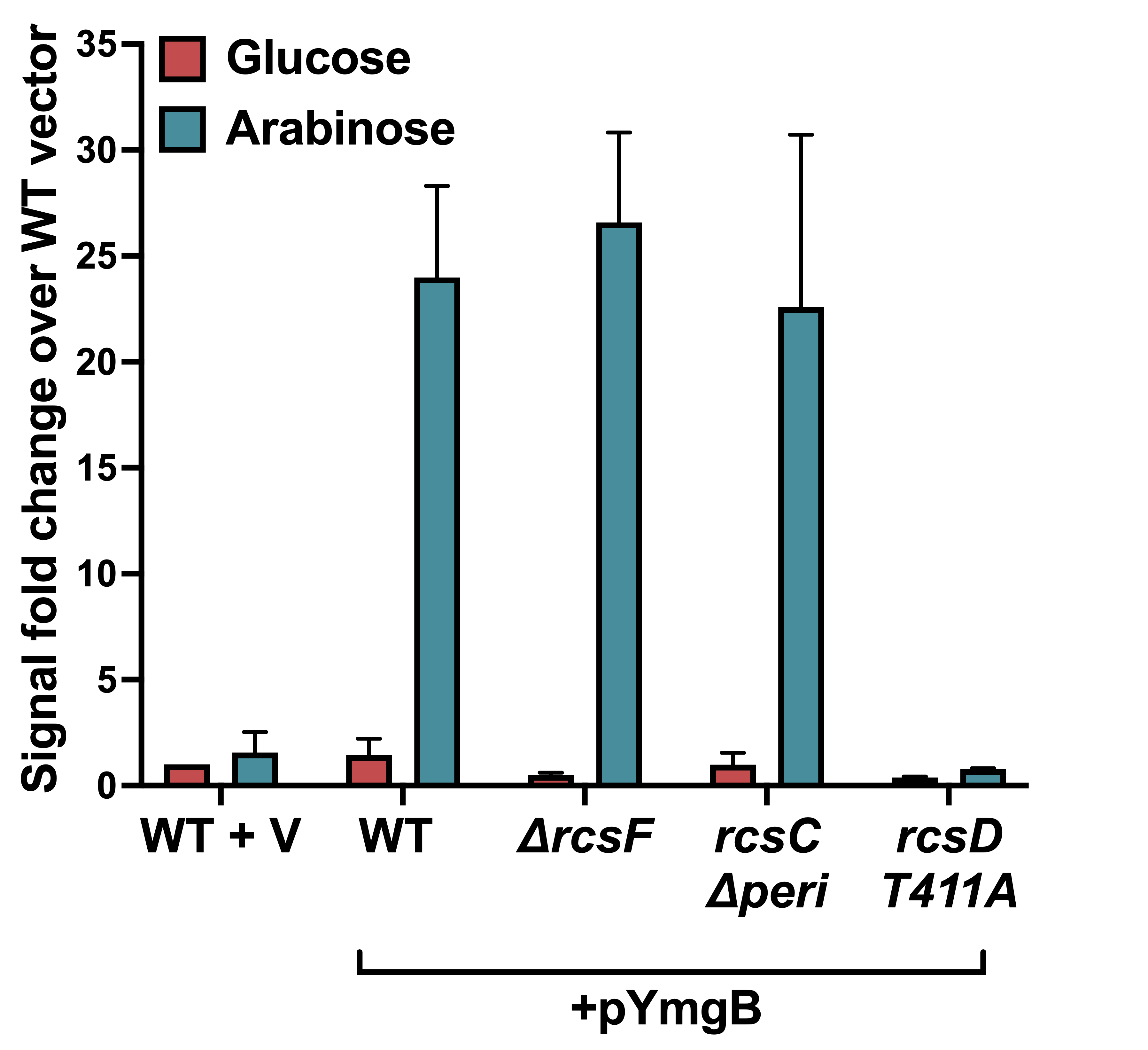

Supplement: S9 Fig — For the PrprA::mCherry assay, strains overexpressing the pBAD33 vector (V) or pBAD33-YmgB (pAP3325) were grown in MOPS minimal glycerol medium containing chloramphenicol (25 μg/ml) and either 0.2% glucose or 0.02% arabinose at 37°C. The RFU at OD 0.4 relative to the uninduced vector control, set to 1, is plotted here. The strains used are: WT (EAW8), rcsF::kan (AP51), rcsCΔperi (EAW70), and rcsD T411A (EAW121). (TIF) [file pgen.1011408.s014.tif]

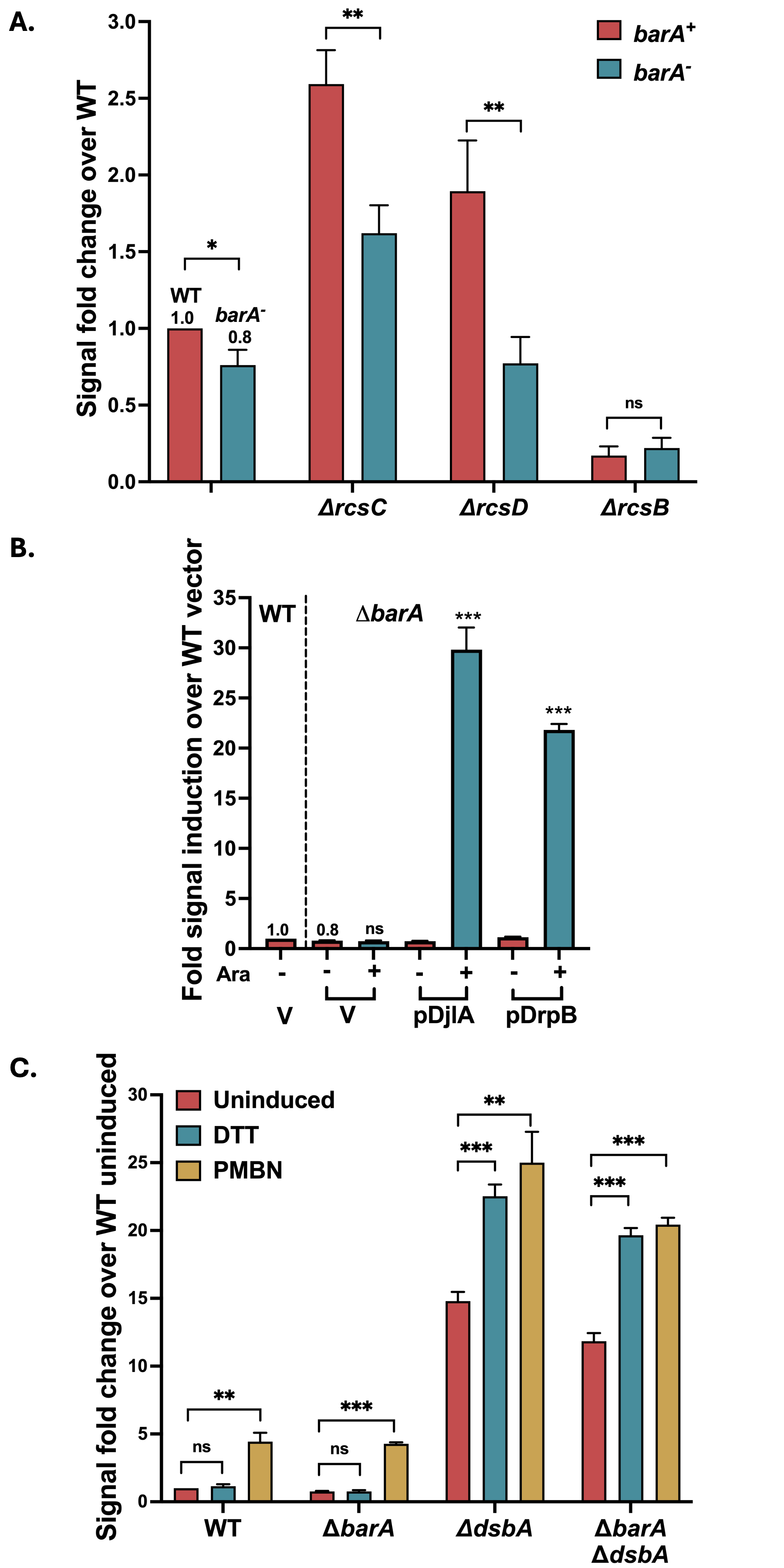

Supplement: S10 Fig — A. Effect of barA deletion on Rcs signaling: For the PrprA::mCherry assay, the cells were grown in MOPS minimal glucose medium at 37°C. The RFU at OD 0.4 as compared to WT, set to 1, is depicted here. The strains used were: WT (EAW8), barA::kan (AP200), ΔrcsC (EAW91), ΔrcsC barA::kan (AP201), ΔrcsD (EAW19), ΔrcsD barA::kan (AP202), rcsB::Tn10 (AP50), and rcsB::Tn10 barA::kan (AP205). B. DjlA and DrpB can activate Rcs in ΔbarA: For the PrprA::mCherry assay, the WT strain (EAW8) and the barA::kan strain (AP200) containing either the vector or plasmids expressing DjlA (pDjlA/pPSG961) or DrpB (pDrpB/pDSW1977) under the control of the arabinose-inducible pBAD promoter were grown in MOPS minimal glycerol medium containing chloramphenicol (25 μg/ml) and either 0.2% glucose or 0.02% arabinose at 37°C. The RFU at OD 0.4 compared to the WT vector uninduced control, set to 1, is plotted. C. DsbA signaling in ΔbarA: For this PrprA::mCherry assay, the strains were grown in MOPS minimal glucose medium at 37°C. The RFU at OD 0.4 as compared to WT uninduced, set to one, is shown here. The cells were subjected to either 1mM DTT or 20μg/ml PMBN. The strains used were: WT (EAW8), barA::kan (AP200), ΔdsbA (AP11), and ΔdsbA barA::kan (AP206). Statistical significance was calculated using multiple unpaired t-tests and is shown as follows: ns (P > 0.05; non- significant), * (P < 0.05), ** (P ≤ 0.01), and *** (P ≤ 0.001). (TIF) [file pgen.1011408.s015.tif]
