# Supplementary material for: RcsF-independent mechanisms of signaling within the Rcs phosphorelay
Source: PLoS Genet. 2024 Dec 26;20(12):e1011408. doi: 10.1371/journal.pgen.1011408 (PMC11709261; doi:10.1371/journal.pgen.1011408)
Supplement: Attachment — Submitted filename: Response to reviews Petchiappan et al 11_15_24.docx [file pgen.1011408.s016.docx]

To the Editor:

We thank all of the reviewers and the editor for the thoughtful and useful reviews. We have responded to the reviews (detailed below, point by point). This includes, in addition to revisions to the text and addition of references at various points, the following other notable changes:

1) We replaced Fig. S4E with a new version (grown under somewhat different conditions), in response to a point raised by Reviewer 1.
2) We added new data in a new supplemental Figure (Fig. S10) at the end of results, testing our factors in the absence of BarA, as requested by Reviewer 1.
3) Primary data files (Prism files) for figures in the paper have been added as SI Data.

**Response to Reviewers**

**Reviewer #1**: In this manuscript, Petchiappan and colleagues unveil three distinct pathways of Rcs activation that are independent of the sensor protein RcsF, that is the mutation of dsbA, the overexpression of the DnaK cochaperone DjlA, and of the inner membrane protein DrpB, using a combination of genetics and phenotypic approaches. The findings of the study undoubtedly advance the global understanding of Rcs signaling, which is rather complex, and provide additional insights on the implication of the different Rcs components and of their interaction in integrating different input signals.

The experiments are properly designed with the proper controls and the conclusions well supported with compelling data. Although the manuscript is quite dense because of the large amount of data, it is very well written and results are well interpreted and analyzed, thereby supporting the proposed model.

I have few comments, mostly minor, and some questions that I recommend the authors to address to improve the manuscript.

**Response:** We thank the reviewer for the careful reading of the paper and the useful questions, which we have attempted to answer.
Comments

1) Introduction.

An RcsF-independent pathway of Rcs activation was identified in Salmonella (Salvail and Groisman, 2020; PMID: 32392214) in which BarA promotes RcsB activation during exponential growth in LB. The authors should mention these findings in the introduction.

**Response**: We have added this to the introduction: “Overproduction of the response regulator RcsB is sufficient to activate downstream targets; work in *Salmonella* identified the phosphorelay protein BarA as an RcsF-independent activator of RcsB.”

2) Results. Lines 166-167. “[…] while deletion of rcsC caused an increase in the basal level of PrprA::mCherry expression […]”

Is the fusion activity increased because of the absence of RcsC’s phosphatase activity? The authors should provide a short interpretation of the phenotype in the text.

**Response:** The text has been modified to explain this explicitly:
“Deletion of *rcsC* or of *rcsD* leads to higher basal levels of expression of the reporter, interpreted as reflecting loss of phosphatase activity combined with some phosphorylation of RcsB from other sources.”

3) Figure S4E.

Why is dsbA mutation not resulting in an increase of fusion activity in the absence of inducer (uninduced IgaA dsbA+ compared with uninduced IgaA delta dsbA)? The strain is delta rcsD but was complemented with a plasmid expressing rcsD, which should recover Rcs signaling.

**Response**: Thank you for pointing this out. In the experiment in Fig. 1C, in which there is an increase in reporter activity in the absence of *dsbA*, cells were grown in glucose minimal media, while for Fig. S4E, cells were grown in LB. Because of the high level of mucoidy and growth defect of the C4S strain (leading to the rapid accumulation of suppressor mutations), we tried to limit growth and therefore inoculated directly from the transformed colonies into LB. In LB, loss of *dsbA* is not sufficient to lead to as high a level of expression, as seen in the original version of this figure panel, without the addition of DTT. We repeated this experiment in LB with glucose; this improves growth and now, as in minimal, loss of DsbA increases basal level of expression. We have replaced the previous Fig. S4E with the glucose-grown version, which is much more similar to the minimal glucose media results. We also emphasize the difference in growth condition in the figure legend: “The resulting transformed colonies were inoculated without further purification in LB medium with glucose (.2%) and Ampicillin, grown at 37°C and their fluorescence monitored. This protocol was used to avoid the accumulation of suppressors seen in the C4S strain during purification and passaging.”

4) Results. Line 368.

What was the goal of doing the assay at high temperature? The authors should briefly explain the rationale of the experiment.

**Response:** This was based on a previously observed phenotype of deletion of *djlA* at high temperature, now explained in the text: “It has been shown that deletion of *djlA* rendered a *dnaJ* deletion strain unable to grow at 40° and above (Genevaux et al, 2001), suggesting an important role at high temperatures. However, even at 42°C, no effect of deleting *djlA* on Rcs basal or induced activity was detected (Fig. S6C). “

5) Figure S6A.

Is DjlA expressed under the tested conditions? If possible, authors should provide evidence from their own data or from the literature that the protein is produced under the growth conditions tested. The absence of phenotype for the djlA mutation could be due to DjlA not being expressed, or not sufficiently, under the conditions in which the experiment was performed. That the djlA mutation has an effect on IgaA-RcsD interaction in Fig. S6C suggests that DjlA is produced, but one must consider that the strain and growth conditions used for the BACTH assay are different than the ones used in Fig. S6A.

**Response:** We do not have direct evidence of our own in terms of the expression level of DjlA under the growth conditions of our assays. While there are synthetic phenotypes seen in the absence of other DnaJ proteins, it is possible that absence of one or more *dnaJ* family genes affects expression of others. We note in the discussion that groups have seen phenotypes of the *djlA* deletion under Rcs constitutive induction conditions (ref. 12, 66); in the Shiba et al paper, there is evidence for DjlA acting negatively on signal strength (measuring a *cps* transcriptional reporter rather than our *rprA* transcriptional reporter), but suggesting a role for single copy DjlA in mediating Rcs behavior. We examined available databases. Ribosome profiling (as summarized in Ecocyc shows relatively low (130 molecules/cell) of *djlA* in MOPS glucose; a compilation of proteomic studies suggests DjlA is a bit above the midpoint for protein abundance (ppm). We now reference the ribosome profiling in the text, and note this as a caveat on our failure to see a single-copy phenotype:

“Based on ribosome profiling, *djlA* is expressed at a modest level at 37°; little is currently known about expression of this protein under other growth conditions. Therefore, we cannot be sure whether DjlA is significantly expressed under these assay conditions. Overall, these results are consistent with the idea that DjlA plays a role in the cytoplasmic signaling interactions of IgaA and RcsD, but under conditions not yet identified.”

6) Figure S8B.

Is DrpB expressed under the growth conditions of the assay? If possible, authors should provide evidence from their own data or from the literature that the protein is produced under the growth conditions tested. The absence of phenotype for the drpB mutation could be due to DrpB not being expressed, or not sufficiently, under the conditions in which the experiment was performed.

**Response**: As with DjlA, we do not have much information or current tools to evaluate the levels of this protein (or what it might do in single copy, definitely a whole study in itself). We included what we know: “Ribosome profiling suggests a modest expression of DrpB from the chromosome, but very little is known about the roles and expression level of the single copy DrpB protein. More studies are needed to ascertain if multicopy DrpB activates the Rcs cascade by interacting directly with RcsC or more indirectly. More broadly, we do not know whether there is a physiological condition during which DrpB made from the single copy gene acts to promote Rcs activation, or whether the multicopy effect triggers a stress that leads to activation, but we would expect a critical role for the RcsC periplasmic domain in either case. “

7) Results

As BarA was previously established as a RcsF-independent activator of RcsB in Salmonella (Salvail and Groisman, 2020; PMID: 32392214), authors should determine whether the Rcs activation phenotypes observed upon DjlA or DrpB overexpression or dsbA inactivation also occur in a barA mutant to rule out whether those are BarA-dependent or not, assuming that BarA also activates RcsB in E. coli.

**Response**: We have added a new supplemental figure (Fig. S10) to address this question. While we did not explore the conditions under which BarA might be most important in *E. coli*, we did see an effect on levels that suggest it is likely to act in *E. coli*, but is not needed for the factors discussed here. We have added a paragraph to the results: “As noted above, the BarA phosphorelay protein was shown to regulate RcsB in Salmonella, independent of RcsF. While our identification of the mode of action of DsbA, DrpB and DjlA would suggest that they act upstream of RcsB, we tested whether BarA was necessary for these factors to induce Rcs. First, the effect of a *barA* mutant on expression of our reporter under normal growth was tested (Fig. S10A). The basal expression of our reporter was modestly reduced in the *barA* mutant, suggesting that, as in Salmonella, BarA may signal to regulate RcsB; a reduction was also seen in strains deleted for *rcsC* or *rcsD*, but not for the *rcsB* deletion, consistent with an effect on RcsB. Overexpression of DjlA or DrpB were still able to induce the reporter in a *barA* mutant (Fig. S10B, compare to Fig. 1B). Similarly, deletion of *dsbA* induced signaling in the absence of *barA* (Fig. S10C). Therefore, these three RcsF-independent mechanisms are also independent of BarA.”

Minor Comments

1) Author summary. Line 41.

Replace “Enterobacteria” with “enterobacteria” to be consistent with the way it is written in the rest of the manuscript.
**Response**: Changed as requested.

2) Results. Line 129.

A reference should be added to support the information provided on Spot42.
**Response:** References added.

3) Results. Lines 144-145.

The statement should be supported by a reference.

**Response**: Rephrased and reference added.

4) Results. Line 247.

Replace “Bordetella” with “Bordetella pertussis”.
**Response:** Added.

5) Results. Line 248.

Add “E. coli” before “strain”.
**Response:** added.

6) Results. Line 281.

Add “data not shown” after “mucoid”.
**Response:** PLOS does not allow statements such as data not shown. No change was made here.

7) Results. Lines 387-388. “DrpB localizes to the divisome and overexpression allowed the ΔftsEX.”

This sentence seems incomplete and should be revised.
**Response**: corrected: “DrpB localizes to the divisome and overexpression allowed the growth of an D*ftsEX* strain at low osmolarity.”

8) Results. Line 438.

Replace “targts” with “target”.
**Response**: corrected.

9) Discussion. Line 545.

Add a space between “by” and “DrpB”.

**Response:** corrected.

**Reviewer #2:** The Rcs stress response is a very complex (the most complex identified so far?) envelope stress response in Gram-negative bacteria. The RcsBCDF-IgaA factors were identified many years ago. Even though significant advances in understanding this system have been made, we still know very little about how signals are sensed and transmitted in the Rcs system. This work by Petchiappan et al. addresses this problem. Using solid, thoroughly applied, classical (yes!) genetic approaches, the authors found that overproduction of the division protein DrpB activates Rcs. They further characterized requirements in the Rcs system for this activation. The authors also characterized other known “signals”, mainly the overproduction of the chaperone DjlA and the loss of the DsbA periplasmic oxidoreductase. In addition to providing mechanistic insights into how these signals are sensed and transmitted, the work is first to show experimental evidence supporting that the RcsC histidine kinase acts as a sensor for at least some signals. Previously, the only bonified sensor in Rcs was the outer membrane lipoprotein RcsF.

This study addresses a fundamental question (how signals are sensed and transmitted) relevant to the bacterial cell envelope, signal transduction, and physiology. Answering this question is often the major limitation in understanding stress responses. The work was very well designed and executed. The authors were very thorough. The manuscript was very well crafted, and the conclusions presented by the authors are justified. It was a pleasure to read. Well done! As a reviewer, I truly appreciate the high quality of the data and manuscript. I only have a minor point that I think the authors should address, as well as two corrections.
**Response**: We thank the reviewer for their positive review (and their appreciation of our genetics).

Minor point:
1) The loss of activation by the DjlA-TM variant could be the result of a loss of protein localization and/or protein stability (maybe the protein is not stable if not anchored to the membrane). This should be clearly stated when discussing the relevant results in the Results and Discussion sections unless the authors can rule out issues with protein levels through immunoblotting (not required to include these data, text clarification would suffice).

**Response**: In previous work by others, a similar construct of DjlA lacking its transmembrane helix was shown to be stable and functional as a cochaperone (Kelley and Georgopoulos, 1997; Genevaux et al., 2001). This is now mentioned in the text with the appropriate references: “We note that others have found that a very similar construct of DjlA lacking the transmembrane region is stable and has co-chaperone function, consistent with our construct showing function in the BACTH assay. “

Text corrections:
Line 252: "We introduced the dsbA null mutant into the host for the BACTH assay" should be "We introduced the dsbA null mutant allele into the host for the BACTH assay".

**Response**: corrected.
Line 406: "DrpB mutants" should be "drpB (italics) mutants”

**Response**: Corrected.

**Reviewer #3**: Regulator of Capsule Synthesis (Rcs) is a complex stress response system involving the RcsCDB phosphorelay and additional regulators, including RcsF and IgaA. Many different conditions induce Rcs, but the signal and the molecular mechanism of activation remain largely unknown. While most conditions require the sensory component RcsF to alleviate phosphorelay inhibition by IgaA, there are known genetic conditions that can lead to activated Rcs even in the absence of RcsF, such as ΔdsbA and DjlA overexpression. In this manuscript, the authors report an additional condition (DrpB overexpression) identified in a screen and characterize all three condition for potential mechanisms of action. They show that ΔdsbA and DjlA overexpression likely interfere with IgaA/RcsD interaction by modulating protein folding, while DrpB overexpression induces Rcs via a different mechanism, likely by generating a signal recognized by the periplasmic domain of the RcsC kinase. Overall, this report adds yet another level of complexity to Rcs and provides evidence for at least some sensory function of the RcsC kinases.

**Response:** We thank the reviewer for the insightful comments on our paper.

Major comments:

My first comment is related to semantics. The authors refer several times in the manuscript to these conditions as distinct “signaling pathways.” Personally, I find this confusing, as the authors show that activation of Rcs is a result of the canonical RcsCDB phosphorelay, so the signal transduction/phosphorylation pathway did not change. It may also be misleading to call them “signals” as it may imply physiological signals for the Rcs system. For example, the authors argue that ΔdsbA results in a misfolded IgaA due to the reduction of disulfides rather than generating a “signal”. Essentially, Rcs activation is a result of malfunction of a Rcs component. As the authors discuss, it is not yet clear whether these conditions are physiologically relevant. So, I think the text should be carefully revised to find a better way to describe the reasons behind changes in Rcs activity.

**Response**: We agree that this is an issue that needs care in how we refer to it. As noted, at this time we don’t have evidence for a signal affecting disulfide bond formation in IgaA, for instance, although we also would not like to fully rule it out in all circumstances. We have gone through the manuscript, editing wording in many cases to say activation of Rcs rather than signaling. In some cases, we have left it as signaling, because that is what we are suggesting (specifically in the role of activation that depends on the RcsC periplasmic domain).

I don’t agree with the conclusion in lines 174-175 that the effect of ΔdsbA is independent of the RcsC periplasmic domain, as deletion of rcsCΔperi in the ΔdsbA background leads to a dramatic reduction of Rcs activity. It is possible that because ΔdsbA is quite pleiotropic, it acts both directly on IgaA but also creates an unknown “signal’ recognized by the RcsC periplasmic domain. Alternatively, there may be some effects of rcsCΔperi on IgaA/RcsD regulation. After all, the authors argue that Rcs signaling can be induced through the RcsC periplasmic domain, and hence must be able to overcome the IgaA/RcsD checkpoint. To this end, is there any effect of rcsCΔperi and ΔdsbA mutations in the igaA C4S background? This is a simple experiment that may help clarify the role of the RcsC periplasmic domain.

**Response**: We agree that there are broader effects of deleting *dsbA* (not surprising, given the pleiotropic effects of this deletion, as the referee notes), and overall the RcsC∆peri does seem to lower activity in ways we don’t yet understand. We carried out the following experiment to look at this, with the results shown here: Starting from an igaAC4S ∆rcsD strain, dsbA::kan was introduced by P1 transduction. Into this, an rcsC::Tn10 insertion was introduced by P1 transduction; this replaces the rcsD deletion as well (so strain in rcsC- rcsD+). This was then transformed with pBAD plasmids expressing either RcsC+ or RcsC∆peri, and the cells were plated on LB Amp plates with glucose (to repress the pBAD promoter). The igaA C4S strain is very mucoid, regardless of the state of *dsbA*. In this context, the rcsC∆peri strain is less mucoid, again with or without *dsbA*.

The results in the (rather complicated) construction above are consistent with the RcsC∆peri reducing signal in the IgaAC4S strain. We were unable to measure rprA-mcherry in these without also blocking mucoidy; we also find that the igaAC4S strain is very unstable, picking up suppressors. We have not added this to the paper, since it doesn’t really provide any new insight, but we changed some of the text to discuss the likelihood that the RcsC∆peri protein has somewhat reduced activity. We have added text to discuss this: “It seems likely that the loss of *dsbA* has additional effects in the periplasm, and this may contribute to the muted response in signal in the *rcsC*∆*peri* strain (Fig. 2A). It is also likely that the RcsC∆peri protein is somewhat perturbed in activity, for instance somewhat reducing the balance of kinase to phosphatase, leading to less activity of the reporter. DjlA overexpression was also able to activate signaling in the *rcsC*∆*peri* strain, although at a somewhat decreased level (Fig. 2B).”

Additional comments:

Line 222: Remove RcsD.

**Response**: Rephrased to clarify: “Aside from RcsF, three other proteins in the phosphorelay (RcsC, RcsD, and IgaA) have a periplasmic domain. However, RcsD does not have any conserved cysteine residues within its periplasmic domain, ruling it out as a direct target of DsbA.”

Lines 360-361: How can DjlA regulate cytoplasmic interaction between IgaA/RcsD if it has an effect on IgaAΔcyto/RcsD strain, in which there is no cytoplasmic interaction?

**Response**: We were somewhat surprised by this result, too. This is why we are proposing that it acts on RcsD, affecting RcsD via the cytoplasmic interaction (consistent with its ability to overcome T411A), and that this interaction then leads to a change in the RcsD/IgaA periplasmic interaction as well (which we know from other work is what drives the BACTH signal). We’ve rephrased some of the text to try to make this clearer: “DjlA was also able to disrupt the interaction of IgaA∆cyt with RcsD (Fig. S5C). We have previously shown that the periplasmic interaction of IgaA and RcsD drives the signal in the BACTH assay, consistent with the high interaction seen in Fig. S5C for IgaA∆cyt with RcsD. Assuming DjlA, with the J-domain in the cytoplasm, interacts with cytoplasmic domains of its substrates, the observation that full-length DjlA can disrupt IgaADcyt-RcsD interactions would suggest that DjlA acts on the PAS-like domain of RcsD, leading to conformational changes that not only overcome the T411A interaction with IgaA but also lessen the strong periplasmic contacts between RcsD and IgaA.”

An alternative explanation for DjlA is that its overexpression may interfere with membrane insertion, for example, of IgaA. This would explain why RcsD T411A does not have an effect in this background. I think there were some reports about DnaK modulated membrane protein transport.

**Response**: We don’t have any information on this possibility. Given the dramatic phenotypes resulting from many mutant alleles, it seems unlikely that anything this drastic is going on. For instance, deletion of the periplasmic domain of IgaA leads to lethality, not suppressed by RcsDT411A (Wall et al, 2020 paper). Possibly the slowed growth when DjlA is overexpressed does affect IgaA or RcsD folding, but given that we think there is another explanation for what is happening here, we have not changed wording in the paper.

Line 490: Oxidative stress typically promotes cysteine oxidation. If anything, it is under anaerobic conditions combined with a decrease in DsbA activity that can lead to disulfide reduction.

Response: Thank you for correcting this; we have not investigated anaerobic growth here, but generalized the comment: “We cannot rule out the possibility that growth conditions exist under which IgaA disulfide bonds might be disrupted sufficiently to induce Rcs signaling. “

I think the manuscript may benefit from some polishing of the writing and the flow. There are some parts that are repetitive. My suggestion is to move the DrpB screen section toward the end, ahead of the DrpB mechanism section, as these sections appear to be disconnected in the current version.

**Response**: We have not moved the sections around. Because we think it is useful to compare the three mechanisms early on in the paper, emphasizing their differences, we prefer to introduce DrpB early. We’ve tried to eliminate some redundancy in the text, although in some cases felt it was necessary to help the reader follow the logic in this complicated system.

There are important statements in the introduction and throughout the paper that don’t have any references.

**Response**: We have added a number of additional references throughout as suggested by other reviewers and by our own rereading.
